# Self-powered bioelectronics for peripheral nerve regeneration: Electroactive materials, energy transduction, and therapeutic mechanisms

**DOI:** 10.1016/j.mtbio.2026.103734

**Published:** 2026-10-08

**Authors:** Fu-Cheng Kao, Monika Pebam, Parag Parashar, Sheng-Fen Wang, Ping-Yeh Chiu, Tsung-Ting Tsai, Chi-Chien Niu, Shih-Heng Chen, Dongwhi Choi, Ming-Kai Hsieh, Zong-Hong Lin

**Affiliations:** a Department of Orthopaedic Surgery, Spine Section, Bone and Joint Research Center, Chang Gung Memorial Hospital, Taoyuan, 33302, Taiwan; b Department of Anesthesiology, Chang Gung Memorial Hospital, Taoyuan, 33302, Taiwan; c Department of Plastic and Reconstructive Surgery, Chang Gung Memorial Hospital Keelung Branch, Keelung, 204, Taiwan; d College of Medicine, Chang Gung University, Taoyuan, 33302, Taiwan; e Department of Biomedical Engineering, National Taiwan University, Taipei, 10617, Taiwan; f Department of Mechanical Engineering (Integrated Engineering Program), Kyung Hee University, 1732 Deogyeong-daero, Yongin, Gyeonggi, 17104, South Korea

**Keywords:** Self-powered electroceuticals, Peripheral nerve regeneration, Bioelectronic medicine, Triboelectric nanogenerators, Piezoelectric nanogenerators, Biodegradable galvanic devices

## Abstract

Peripheral nerve injuries remain difficult to treat because axonal regeneration is slow, incomplete, and highly dependent on timely biochemical, structural, and electrical cues. Self-powered electroceuticals offer an emerging route to deliver localized electrical stimulation with reduced dependence on bulky batteries and percutaneous leads. This review summarizes recent advances in self-powered bioelectronic systems for nerve repair, with emphasis on three representative platforms: self-electrified galvanic devices, triboelectric nanogenerators, and piezoelectric nanogenerators. From a materials and interfacial engineering perspective, we discuss how redox reactions, contact electrification, and piezoelectric polarization convert physiological or externally applied energy into therapeutic electrical outputs. Key design factors, including biodegradable metals, electroactive polymers, piezoelectric composites, conductive interfaces, encapsulation strategies, and nerve-guidance architectures, are critically examined in relation to output stability, degradation behavior, tissue compatibility, and regenerative efficacy. We further highlight preclinical evidence that self-powered electrical cues influence Schwann cell activity, axonal elongation, neurotrophic factor secretion, immune modulation, and downstream signaling pathways to enhance myelination, suppress muscle atrophy, and improve functional recovery. Finally, current limitations and future opportunities are discussed, including stimulation-dose standardization, degradation-byproduct management, scalable fabrication, closed-loop integration, and large-animal validation. This review provides a unified-materials–interface–device–biology framework for developing clinically translatable self-powered electroceuticals for nerve regeneration.

## Graphical abstract

Self-powered bioelectronics enable autonomous electrical stimulation for peripheral nerve regeneration through bioresorbable galvanic devices, triboelectric nanogenerators, and piezoelectric nanogenerators. By integrating electroactive materials, energy transduction, and bioelectronic interfaces, these platforms promote Schwann cell activation, axonal regrowth, remyelination, and functional recovery.

**Figure:**
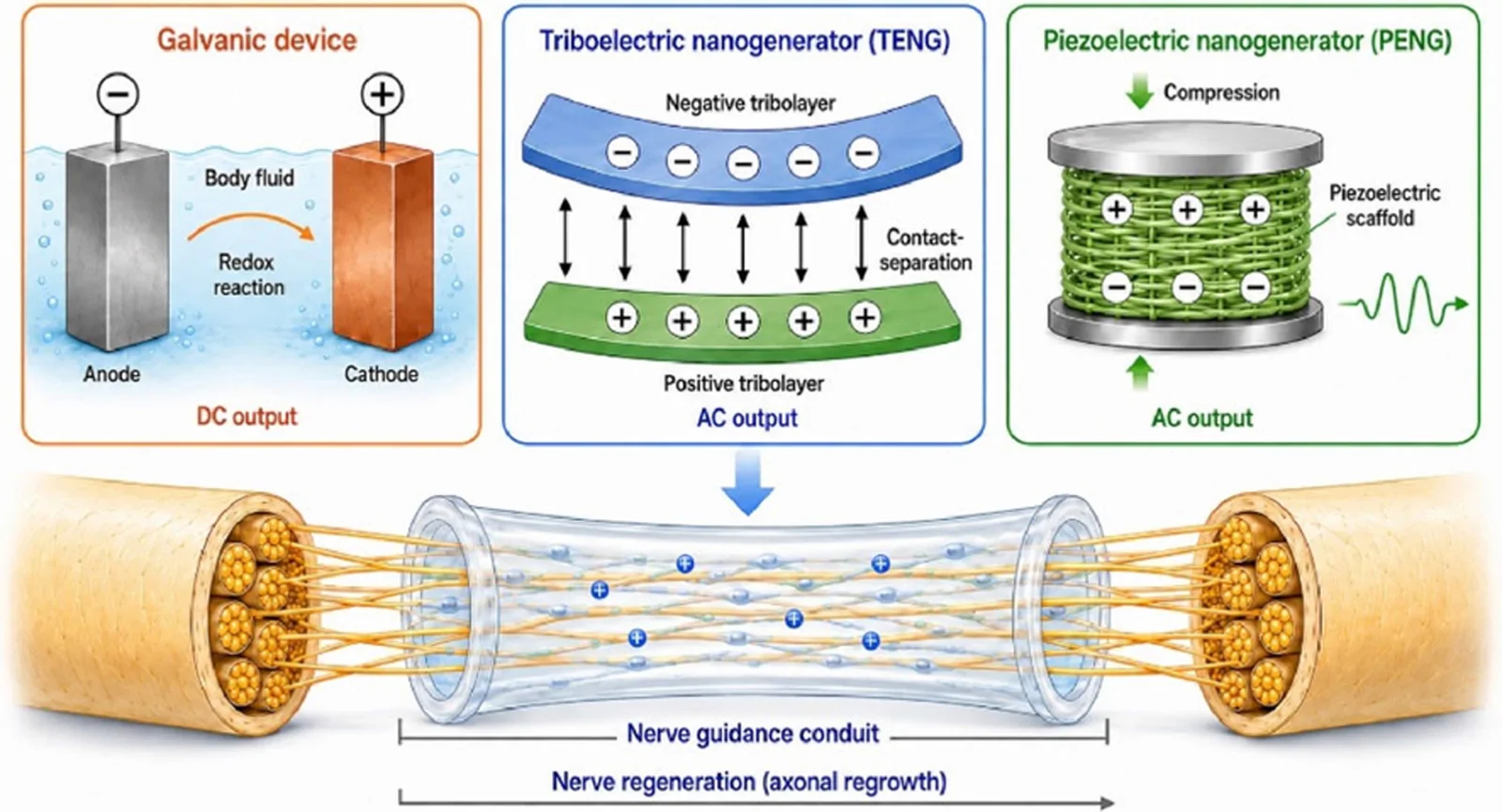

## 1 Introduction

The peripheral nervous system (PNS) serves as an indispensable communication network, transmitting motor commands, relaying sensory information, and regulating autonomic functions between the central nervous system and effector organs [1]. Despite its functional sophistication, peripheral nerve tissue is profoundly vulnerable to traumatic, compressive, and ischemic insults. Peripheral nerve injuries (PNIs) represent one of the most debilitating categories of neurological trauma, with an estimated annual incidence of 13–23 per 100,000 individuals, often resulting in devastating sensory deficits, motor paralysis, and chronic neuropathic pain [[1], [2], [3], [4], [5]]. Trauma and other insults can disrupt peripheral nerve structure and function [6]. Following transection or severe crush injury, the distal axonal segment undergoes Wallerian degeneration, an orchestrated cascade in which the cytoskeleton disassembles and Schwann cells (SCs) transition into an activated repair phenotype. These repair SCs proliferate and align within residual basal lamina tubes to form “Bands of Büngner" that guide regenerating sprouts, while recruited macrophages help clear axonal and myelin debris [7,8]. However, axonal regrowth proceeds at approximately 1–3 mm per day; protracted regeneration often leads to SC phenotype loss, tube collapse, and irreversible muscle atrophy. Recovery remains variable even after surgical repair [3]. The consequences of severe peripheral nerve injury result in formidable challenges due to chronic pain, such as neuroma formation [9]. Early diagnosis is crucial to monitor healing stages after severe neuropathic injury, which provides key physiological and pathological insights for timely medical interventions. To address these challenges, multifunctional peripheral nerve interfaces that employ bioreabsorbable electronic platform materials facilitate nerve regeneration and pain management. This biodegradable nerve interface relies on its flexibility integrated with wireless technology for signal amplification, digitization, and remote transmission, potentially reducing the need for removal surgery when whole-device bioresorption is established.

Non-neuronal cells contribute to recovery after PNS injury. Schwann cells release numerous growth factors like nerve growth factor (NGF) and glial cell-derived neurotrophic factor (GDNF) for nerve regeneration. On the other hand, macrophages help to clear debris from myelin and axons, while endothelial and fibroblast cells maintain the structural integrity of the peripheral nervous system. Repair Schwann cells aligned in Bands of Büngner provide extracellular matrix (ECM) proteins and neurotrophic factors that support axonal regeneration. The axonal sprouts regrow, and SCs remyelinate, helping to restore nerve impulse conduction, which is crucial for functional peripheral nerve recovery [7,8]. Nonetheless, the slow rate of nerve regeneration and functional recovery with persistent motor or sensory deficits leads to progressive muscle fiber atrophy and permanent functional disabilities. As an alternative, artificial nerve graft regeneration employing nanosystems offers a promising therapeutic approach.

The clinical gold standard for bridging nerve gaps remains the autologous nerve graft, typically harvested from expendable sites such as the sural nerve [2,3]. Although autografts provide a native scaffold with viable SCs and essential neurotrophic factors, their utility is fundamentally constrained by limited donor availability, donor-site morbidity, and potential size mismatches. To circumvent these limitations, synthetic and biological nerve guidance conduits (NGCs) composed of collagen, polyglycolic acid (PGA), or poly(lactic-co-glycolic acid) (PLGA) have been developed. However, hollow NGC performance depends on gap length, nerve type, and injury context, and outcomes in long defects remain challenging. These passive conduits offer structural guidance but fail to deliver the dynamic biochemical and bioelectrical cues essential for directing rapid axonal elongation, maintaining SC activity, and preventing denervation-induced muscle atrophy [3,10].

A growing body of evidence demonstrates that exogenous electrical stimulation (ES) can enhance nerve regeneration by amplifying endogenous “injury currents” that direct reparative cell migration via galvanotaxis [2,5]. Mechanistically, ES upregulates regeneration-associated genes (RAGs) such as GAP-43, enhances BDNF/TrkB signaling through calcium-dependent cAMP signaling, and activates downstream PI3K/Akt and MAPK/ERK cascades implicated in sprouting and neurite outgrowth. Clinical studies support brief perioperative ES (e.g., 1 h at 20 Hz) to enhance selected reinnervation outcomes; improved electrophysiology does not necessarily translate into improved functional recovery [11,5]. ES is categorized into direct current (DC), alternating current (AC), capacitive coupling (CC), and pulsed electromagnetic field (PEMF or inductive coupling) mechanisms. For direct coupling methods, DC or AC electrodes will be directly inserted or implanted for electrostimulation inside the body, whereas indirect coupling methods, CC or PEMF, induce electrical stimulation within the cells exogenously by generating a homogeneous electromagnetic field between electrodes or converting the electromagnetic field into pulse stimuli to mimic the body's electrophysiological rhythm [12]. At the cellular level, exogenous or self-powered electrical cues influence neuronal membrane polarization, thereby promoting intracellular Ca^2+^ influx through voltage-gated calcium channels, and may affect downstream signaling pathways including PI3K/Akt, MAPK/ERK, focal adhesion kinase (FAK), and Yes-associated Protein (YAP) mechanotransduction. The ion-influx signaling pathway enhances neurite extension, SC proliferation, cell migration, neurotrophic factor secretion, and context-dependent macrophage responses [11,13,14]. However, conventional delivery systems rely on externally powered electrodes with percutaneous leads or bulky implantable pulse generators (IPGs) housing rigid, non-degradable batteries. These systems carry significant risks of infection and chronic inflammation, while necessitating invasive secondary surgeries for device removal [15,16]. These formidable obstacles have catalyzed the development of self-powered approaches capable of delivering localized electrical cues within the biological environment [17]. ES cues regulate cellular metabolism by promoting tissue regeneration and functional recovery post-traumatic injuries. Electroactive biomaterials, such as piezoelectric materials, triboelectric nanogenerators, and conductive polymers, transduce mechanical forces into electrical outputs or deliver localized electrical stimuli, thereby enhancing blood flow and protein synthesis to induce angiogenesis and modulate inflammation [18]. ES modulates neuronal activity through metabotropic receptors and intracellular signaling mechanisms, triggering muscle contraction, gene expression, and synaptic reflex actions to improve cellular activity and tissue connectivity [19].

Over the past decade, the field of neuroengineering has witnessed a decisive paradigm shift from passive structural bridging to active, self-powered bioelectronic neuromodulation. This evolution leverages advanced materials science and electrical engineering to develop transformative treatments, such as spinal cord, vagus nerve, and sciatic stimulation, offering novel solutions for neurological disorders. Central to this progress is the emergence of converging technological strategies: first, self-electrified, fully biodegradable devices (e.g., Mg-FeMn or Zn-Mo galvanic cells) that provide sustained postoperative cues to promote SC repopulation without secondary surgery [9,[20], [21], [22]]; and second, energy-harvesting nanogenerators, including triboelectric (TENGs) [[23], [24], [25], [26]] and piezoelectric (PENGs) [[27], [28], [29], [30], [31]] systems, which convert biomechanical or acoustic energy into on-demand therapeutic fields (Fig. 1). While other strategies like enzymatic biofuel cells have been explored, their neural applications remain limited by enzyme instability and low power density [32,33]. Existing reviews discuss piezoelectric polymers in nervous-tissue regeneration, TENG-mediated therapeutic stimulation, and implantable self-powered systems more broadly [6,34,35].

**Fig. 1:**
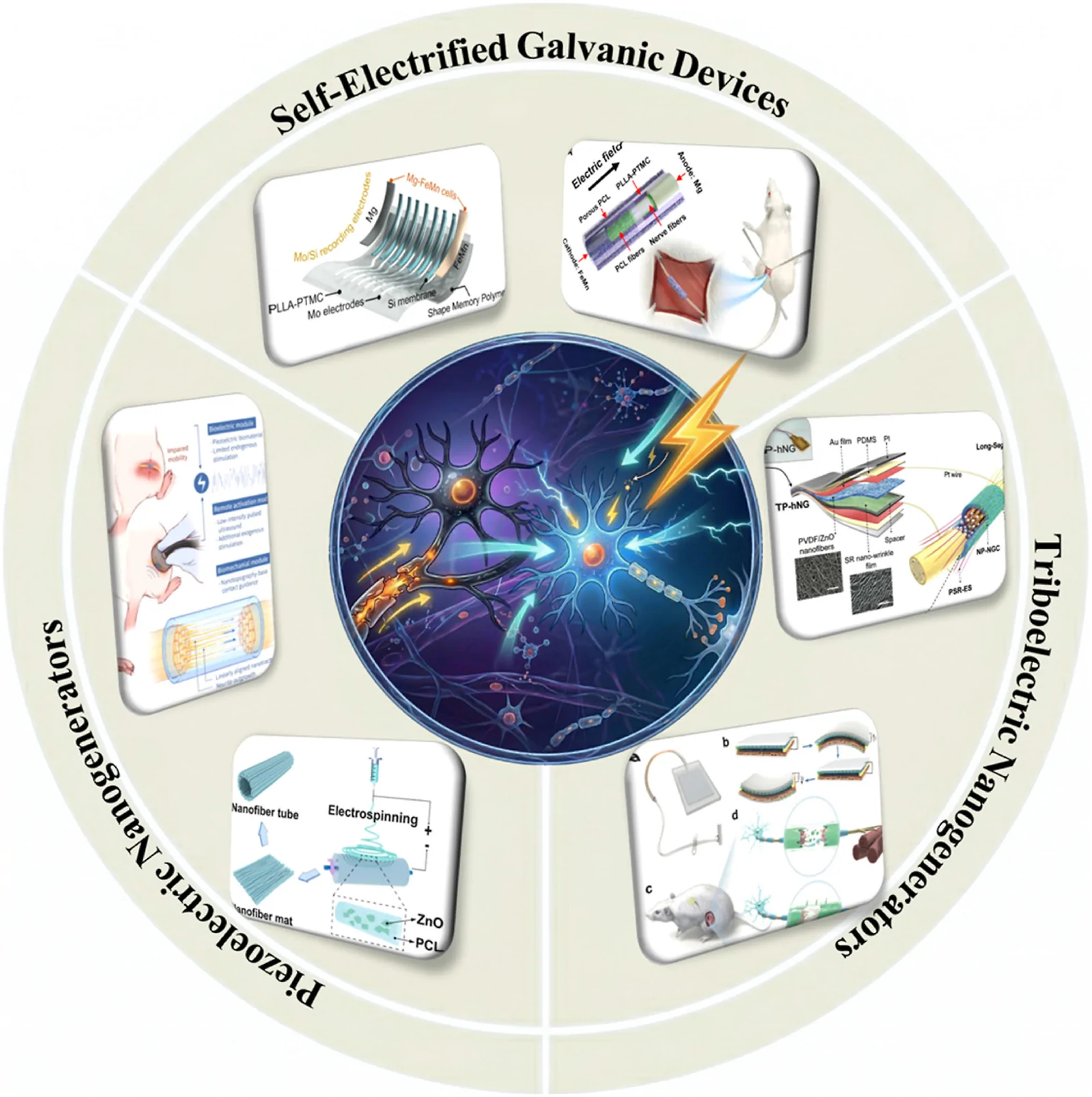
Three self-powered strategies for nerve regeneration: self-electrified galvanic devices (Reproduced with permission [9,20]. Copyright © 2020, AAAS; Copyright © 2025, Springer Nature), triboelectric nanogenerators (Reproduced with permission. Copyright © 2020 and 2021, John Wiley and Sons) [9,20] and piezoelectric nanogenerators (Reproduced with permission. Copyright © 2022 and 2023, ELSEVIER) [36,37].

The present review provides a comprehensive analysis of these converging approaches to electroactive nerve repair, systematically examining their classification: material selection, device design, mechanisms of action, and the current preclinical evidence landscape.

The novelty of this review lies in providing a comprehensive translational framework for self-powered bioelectronics in peripheral nerve regeneration. Beyond summarizing recent advances, a critical and focused comparison of self-electrified galvanic, triboelectric, and piezoelectric platforms specifically for peripheral nerve repair remains limited. It critically examines these three approaches through a common material: interface, device, and biology framework, covering their energy-transduction mechanisms, material and device design, electrical stimulation characteristics, and preclinical evidence for nerve regeneration. Particular emphasis is placed on how the energy source and device architecture determine the nature, duration, and controllability of the electrical stimulus delivered, and how these factors relate to biological responses and functional outcomes. Rather than comparing platforms primarily by unloaded voltage or other isolated device-level metrics, we consider stimulation dose, experimental conditions, regenerative endpoints, degradation behavior, safety, and translational requirements. Through this framework, the review aims to distinguish demonstrated regenerative effects from device-level performance claims and identify the key considerations for developing clinically relevant self-powered electroactive platforms for peripheral nerve repair. The clinical value of these platforms depends on establishing the delivered stimulation dose, long-term safety, and the reproducibility of functional benefit. Its specific contribution is to connect energy dependence and device design to the strength of clinical regenerative evidence and the requirements for translation, rather than ranking technologies by unloaded voltage.

## 2 Self-powered bioelectronics for neuroengineering

The evolution of neuroengineering has been fundamentally reshaped by the synergy between sophisticated biomaterials and self-powered bioelectronic systems, which not only enhance operational efficiency but also ensure the high degree of biocompatibility required for implantable and wearable interfaces [24,27,38]. A self-powered system is an integrated device that operates without an external power source by harvesting energy from ambient sources such as heat, light, or mechanical motion. Traditional methods of delivering electrical stimulation for nerve repair are often hampered by their reliance on external power sources or bulky, non-degradable implanted pulse generators, presenting significant clinical challenges such as infection risks from percutaneous leads, chronic foreign-body responses, and the inevitable need for invasive secondary surgeries [39,40]. To address these drawbacks, a suite of convergent electroactive technologies has emerged over the last decade, categorized by their distinct energy-harvesting modalities, including self-electrified galvanic devices, triboelectric and piezoelectric nanogenerators. These systems utilize diverse energy sources, ranging from mechanical motion to electrochemical reactions, to provide localized electrical power required for applications extending from tactile sensing to the sophisticated restoration of damaged neural pathways [20,39,41]. Implantable biodegradable self-powered bioelectronic devices have achieved remarkable potential towards the treatment of neurological disorders and peripheral nerve injuries by stimulating controlled electrical guidance to regenerate neuronal activity, enhance axonal regeneration, and promote functional recovery. The subsequent sections provide a systematic classification of these technologies, detailing their fundamental working mechanisms and the critical design principles governing material selection for effective nerve regeneration.

Here, “self-powered” is used as an umbrella term encompassing three distinct energy categories, each based on its degree of autonomy. Autonomous galvanic devices consume stored electrochemical reactants or mechanical processes and use physiological fluid as an electrolyte to generate spontaneous redox reactions between electrodes [42]. Biomechanically powered TENGs/PENGs harvest energy from physiological activities (respiration, movement, or local deformation), so their output depends on activity [43]. Externally activated battery-free systems, TENGs/PENGs, require mechanical energy sources such as ultrasound, shockwaves, or low-frequency vibration. Importantly, while all three platforms avoid implanted batteries, only the first category is truly autonomous; the latter two rely on exogenous activation and are therefore not strictly self-powered. For instance, ultrasound-driven biodegradable PENGs and vibration-activated piezoelectric conduits both enhance nerve repair by converting external acoustic or mechanical energy into localized electrical stimulation, yet they remain dependent on external sources. Wireless describes the absence of a transcutaneous connection, whereas “bioresorbable” concerns the complete degradation of the entire implanted device [44]; neither battery-free operation nor partial degradation establishes whole-device resorption. Henceforth, differentiating the autonomous self-powered categories further requires a more accurate description of the energy autonomy that will enable the translational relevance of potent engineered biodegradable bioelectronic implants. These distinctions apply throughout Section 2, which covers materials selection and device design, and throughout Section 4, which discusses its therapeutic applications. The review further discusses comparison studies of galvanic, TENGs, and PENGs devices, respectively, corresponding to nerve repair mechanisms from the existing literature in Table 1, Table 2, Table 3, Table 4, Table 5.

**Table 1:** Comparative mechanisms of electroactive strategies for nerve repair.

Category	Self-Electrified Galvanic Devices	Nanogenerators (PENG/ TENG)
**Energy Source**	Electrochemical redox reaction between dissimilar metal electrodes (Mg–FeMn, Zn–Mo, Zn–O_2_) using body fluids as the electrolyte	Mechanical-to-electrical transduction via piezoelectric material deformation (PENG) or triboelectric contact-separation (TENG)
**Electrical Output**	Continuous DC; 20–250 mV mm^−1^ in the Mg–FeMn study; loaded exposure depends on geometry and tissue conditions	Pulsed or alternating signals synchronized with physiological motion or externally triggered by ultrasound/shockwave; inherently intermittent
**Primary Signaling Pathways**	Reported calcium responses and increased NGF/BDNF/VEGF expression; pathway causality requires study-specific validation	Platform-specific associations with GAP43, RAS/MAPK, and PI3K–Akt; immune-signaling findings are not universal mechanisms
**Structural Guidance**	Can be integrated with aligned fibers, as in Zn–Mo/PCL and Si–Mg/PCL scaffolds	PENG nanofibers simultaneously provide aligned topographical guidance and bioelectric stimulation within the same conduit wall
**Dose Controllability**	Fixed post-implantation; output determined by electrode material and geometry	Tunable by material composition, device architecture, and mechanical input; ultrasound-driven systems allow non-invasive external programming
**Degradation & Byproducts**	Device-dependent resorption; Mg may generate H_2_; local Zn^2+^ exposure and retained cathode/scaffold components require assessment	Bioresorbable variants available (PLGA, PCL, silk); degradation rate engineered to match healing timeline; select platforms support on-demand ultrasound-triggered disintegration
**Potential clinical fit (not established superiority)**	Acute/immobile postoperative phase where continuous, movement-independent stimulation is required	Active rehabilitation phase; or externally activated paradigms for immobilized patients

**Table 2:** Comparison of galvanic-cell-based self-powered devices for nerve repair.

Category	Wang et al. (2020) [20]	Li et al. (2023) [22]	Yu et al. (2024) [21]	Wang et al. (2025) [9]	Wang et al. (2026) [45]
**Device/ System**	Mg-FeMn galvanic cell integrated into NGCs (electrode surface area, 1 cm^2^)	Tubular Zn-O_2_ battery wrapped around nerve segment	Zn-Mo galvanic cell integrated into NGCs with aligned PCL fibers	Bio-Restor: Mg-FeMn galvanic cell + Mo/Si neural recording array	SESS, Si–Mg galvanic cell
**Electrode Composition**	Anode: Mg (3.5 μm) Cathode: FeMn alloy (1.5 μm)	Anode: Zn; cathode: CNT/Pt catalyst for oxygen reduction	Anode: sintered Zn particles Cathode: Mo particles	Anode: Mg (3.5 μm, 1.5 × 5 mm) Cathode: FeMn alloy (1.5 μm, 1.5 μm, 1.5 × 5 mm) Recording: 7-channel Mo/Si array	Mo/Si semiconductor electrodes integrated with an electrochemically active Mg/PCL layer
**Electrical Output**	Scanning rate: 0.2 mVs^−1^ and potential: ± 0.25 V, Electrolyte: PBS body fluid 1.5 S m^−1^	231.4 mWh cm^−3^ in vivo; electrode volume 0.86 mm^3^ (anode + cathode basis)	Output voltage: ∼0.5 V; operation ∼50 h under reported conditions (∼6× the compared Mg system)	DC electric field: 20–250 mV/mm Recording operational: 5.5–8.5 weeks	∼400 mV initial voltage, decreasing gradually to ∼50 mV after 90 days in vivo. Maximum power output: ∼0.15 μW; electrical impedance: ∼200 kΩ.
**Scaffold/ Structural Design**	Bioresorbable NGC with electrospun PCL fibers	Ultra-compact tubular design Direct nerve wrapping	Aligned PCL nanofibers for topographical guidance	Flexible, bioresorbable neural interface Conformal nerve-wrapping design	Electrospun aligned PCL fibrous scaffold, axonal guidance
**Animal Model**	10-mm rat sciatic nerve defect (12-week follow-up)	10-mm rat sciatic nerve defect; 12-week follow-up	10-mm rat sciatic nerve defect (12-week follow-up)	10-mm rat sciatic nerve defect (follow-up to ≥12 weeks)	10 mm rat sciatic nerve defect model
**Key In Vitro Findings**	↑ Calcium activity & neurite outgrowth in DRG neurons (p < 0.01) ↑ Schwann cell proliferation ↑ NGF, BDNF, VEGF expression	Biocompatibility validated Stable electrochemical performance confirmed	↑ Schwann cell proliferation ↑ ATP production	↑ Axonal regrowth markers↑ Regeneration-associated gene expression Accelerated Wallerian degeneration	Enhanced cell migration under electrical stimulation (scratch area reduced to 0.31 ± 0.03 mm^2^ vs control 0.39 ± 0.03 mm^2^), increased, promoted directional PC 12 neurite extension
**Key In Vivo Results**	SFI: −41.51 ± 5.47 (vs. −69.82 ± 3.06 hollow conduit) ↑cMAP amplitude (p < 0.05) ↑ Muscle wet weight ratio Comparable to autograft at 12 weeks	Significant regeneration of long-segment sciatic nerve defects ↑ Nerve fiber density and maturation	Axon length at 3 weeks: 8.43 ± 0.44 mm Fiber diameter: 5.33 ± 1.55 μm (p < 0.05) Myelin thickness: 1.30 ± 0.30 μm (p < 0.001) CMAP: 4.74 ± 1.35 mV at 12 weeks SFI comparable to autograft	↑ Axon density vs. controls (p < 0.001) at week 3 Hindlimb movement observed at week 3 (absent in controls) Real-time neural recording for 5.5–8.5 weeks	SFI −50.38 ± 1.41 at 12 weeks, restored nerve conduction CMAP: 4.19 ± 0.05 mV, Upregulated axon guidance, PI3K–Akt, calcium signaling, ECM interaction, and anti-inflammatory pathways
**Unique Feature/ Novelty**	First fully bioresorbable self-electrified NGC; establishes galvanic-cell self-powering concept for nerve repair	Compact tubular power source; CNT/Pt and PPy persistence precludes a blanket whole-device resorption claim	Zn anode eliminates excess H_2_ generation of Mg systems; aligned PCL fibers add topographical cue	Combined stimulation and neural recording; a basis for future closed-loop control, not demonstrated automatic dose adjustment	First fully biodegradable Si–Mg galvanic bioelectronic scaffold enabling long-term, adaptive, self-powered electrical stimulation using body fluids as the electrolyte

NGC, nerve guidance conduit; NGF, nerve growth factor; BDNF, brain-derived neurotrophic factor; VEGF, vascular endothelial growth factor; cAMP, cyclic adenosine monophosphate; ATP, adenosine triphosphate; CMAP, compound muscle action potential; SFI, sciatic functional index.

**Table 3:** Comparison of TENG-based self-powered systems for nerve repair.

Category	Zheng et al. (2016) [23]	Jin et al. (2021) [18]	Zhou et al. (2022) [24]	Lee et al. (2023) [25]	Niu et al. (2025) [46]	Cai et al. (2026) [26]
**Device name**	BD-TENG (Biodegradable TENG)	TP-hNG (Tribo/piezoelectric hybrid nanogenerator)	CS-TENG (Contact-separation TENG)	ACT-TENG (Acoustically-triggerable transient TENG)	SF- TENG	H-SiO_2_-TENG (Mesoporous silica charge collector)
**Core triboelectric materials**	PLGA, PCL, PVA, PHB/V polymer combinations	Silicone rubber nano-wrinkle film/PVDF,ZnO nanofibers	PDMS/PA6 + Silver nanowires	PHBV/PEG:ChCl film	Silk fibroin, LiCl	PDMS doped with mesoporous silica (H-SiO_2_)
**Electrode Material & Encapsulation**	Mg film (50 nm)	Au film	Aluminum (Al) foil, PTFE and Ecoflex encapsulation	Mg electrodes	Mg electrode	Aluminum (Al) foil, Polyimide
**Driving Mechanism**	Biomechanical motion (simulated 1 Hz low-frequency motion)	Respiratory motion of thorax subcutaneous adipose layer (piezo/tribo synergistic effect)	Biomechanical energy from rat respiration and daily activities	External ultrasound: 20 kHz, 0.5 W cm^−2^; 5 min/day for 5 days in the repair protocol	Vertical contact–separation mode TENG	Biomechanical pressing and separation
**Electrical Output Performance**	∼40 V open circuit; ∼1 μA short circuit (different loading conditions)	2.7 ± 0.2 V/ 0.12 ± 0.2 μA (active state); 2.2 ± 0.15 V, 0.1 ± 0.1 μA (calm state)	≈11.50 ± 0.50 V (voltage); 0.15 ± 0.50 μA (current)	7.8 V peak at 40 MΩ; 60.0 μA peak at 1 Ω (not simultaneous paired measurements)	≈11.50 ± 0.50 V (voltage); 0.15 ± 0.50 μA (current)	Up to ∼8 V
**Device Lifespan & Degradation**	Passive natural degradation: Based on material hydrolysis (e.g., PLGA disintegrates within 40-50 days)	Long-term stable: Maintains effective electrical output 12 weeks post-implantation	Long-term stable: Non-degradable design, multi-layer waterproof encapsulation for stability	High-intensity ultrasound (≥3.0 W cm^−2^) triggers fragmentation; subsequent clearance must be assessed separately	Fully biodegradable, with substantial degradation in 6 weeks	Long-term stable: No obvious decline after 10,000 cycle tests
**Biological target and nerve model**	Directed nerve cell growth, power supply for short-term implantable medical devices	Long-segment sciatic nerve defect (15 mm gap), nerve guide conduit repair, neovascularization	Sciatic nerve crush injury	Peripheral nerve compression injury, CMT1A hereditary neuropathy	Peripheral nerve injury and denervation-induced muscle atrophy	Peripheral nerve injury repair, maintenance of cell resting membrane potential
**Core Technological Advantages**	Composed entirely of bioresorbable materials, eliminates the need for secondary removal surgery	Physiologically self-regulated ES, output signals fully synchronized with respiratory vital capacity	High stability and anti-corrosion encapsulation, silver nanowires enhance surface charge density	Features on-demand lifespan control, provides high-frequency sinusoidal stimulation	Fully biodegradable, biocompatible, self-powered, high-output, fast-response and implantable bioelectronic system	Utilizes deep potential traps of H-SiO_2_ for charge storage, stabilizing

**Table 4:** Comparison of motion-responsive PENG scaffolds and their activation sources.

Category	Cheng et al., 2020 [30]	Mao et al., 2022 [37]	Stratton et al., 2023 [31]	Zhang et al., 2023 [47]	Chen et al., 2025 [43]	Li et al., 2026 [48]
**Material/ Architecture**	PVDF/PCL 3D porous conduit	PCL/ZnO electrospun conduit (PZNF)	Aligned PCL/ZnO electrospun scaffold	SF/PVDF-HFP/MXene electrospun conduit	(PCL)- β-Gly composite nanofibers	PVDF/LM-ZnO + PTNG hybrid conduit
**Fabrication**	Cast/ annealing-solvent displacement (55 °C)	Electrospinning (2 wt% ZnO)	Electrospinning (0–2 wt% ZnO); >90% fiber alignment	Electrospinning (3 wt% MXene)	Electrospinning	Electrospinning + hydrothermal ZnO growth
**Key Piezoelectric Output**	d_33_ = 13.2 p.m. V^−1^; 12× vs. PCL	40–120 mV @ 0.3 MΩ	Piezoelectric + Zn^2+^ release (<6% over 4 wks)	100 mV (vs. 20 mV baseline)	d_33_ up to 11 p.m. V^−1^; bench-film Voc ∼1.5–4.0 V and Isc ∼1–13 nA at 1–10 N. 30 kV is the electrospinning voltage.	d_33_ = 4.6 pC N^−1^ (∼5.1-fold vs 0.9); ∼2 V from the separate PTNG hybrid generator
**Mechanical Input Source**	Cell traction forces	Ambulatory/ Flexcell mechanical loading	Minute mechanical deformation	Animal locomotion (exercise-activated)	External massage-gun vibration: 15 Hz, 30 min every other day for 2 weeks; not autonomous physiological activation	Multi-modal physiological motion
**Animal Model (defect)**	Rat sciatic, 15 mm, 4 months	Rat sciatic, 10 mm, 4 weeks	In vitro only (SC–DRG co-culture)	Rat sciatic, 10 mm, 12 weeks	Rat sciatic nerve defect, 10 mm; 12-week follow-up	Rat sciatic, 8 weeks
**Selected functional or histological outcome**	SFI: −27.7 (dimensionless); NCV: 35.5 m s^−1^	SFI ≈ bridging in situ	Axon length +30% vs. control	SFI: −33.3 ± 3.8 (≈ autograft −36.2)	S100-positive area: 26.9% vs 28.9% autograft; these percentages are not SFI	SFI: −21.2 ± 3.1 (vs. −55.0 suture-only)
**Key Signaling/ Mechanism**	-mediated angiogenesis; axonal myelination	GRB2↑; RAS/MAPK pathway↑	Trk signaling (Zn^2+^-mediated neurotrophin upregulation); contact guidance	SC GFAP/S100/NGF upregulation; antibacterial (>90%)	Neural differentiation; CMAP recovery; myelination	NT-3↑; MBP/NF200/Tuj1↑; surpasses external ES

SFI, sciatic functional index (dimensionless); NCV, nerve conduction velocity; GRB2, growth factor receptor-bound protein-2; SC, Schwann Cell; DRG, Dorsal Root Ganglion; MBP, Myelin Basic Protein.

**Table 5:** Comparison of wireless externally-activated PENG.

Category	Wu et al. (2022) ] [44]	Pi et al. (2023) [36]	Tai et al. (2023) [27]	Xu et al. (2024) [49]	Jeon et al. (2025) [50]	Ding et al. (2025) [28]	Dai et al. (2026) [29]	Qi et al. (2026) [51]
**Piezoelectric** **Material/ Scaffold**	PHBV/PLLA/KNN nanowires (50 wt%) + Mg electrodes PLA/PCL encapsulation	PCL/PVDF aligned nanotracts (d_33_ = 14.07 p.m./V)	P(VDF-TrFE) electrospun nanofiber conduit (500 nm)	BTNPs/P(VDF-TrFE) nanofibers (7 wt%) + pNIPAM/GelMA hydrogel (NGF-loaded)	PLLA/PEG (80:20) electrospun APNF 4-channel NGC	Ti_3_C_2_T_x_/UiO-66 in PLLA scaffold (laser additive mfg.)	PRSC: Rochelle salt in PLLA nanofibers (uniaxial compression) PLCL encapsulation	Methanol-treated silk fibroin (SF)-based Janus membrane, with an oriented GelMA-tannic acid (GT)
**Activation Method** **& Parameters**	Treatment: 100 kHz, 0.3 W cm^−2^; 10 ms pulses at 1 s intervals. Characterization/monitoring: 0.7 W cm^−2^.	LIPUS 1 MHz, 0.5 W/cm^2^ 20% duty cycle	Extracorporeal shockwave 4 bar, 1000 pulses x 2/week	Ultrasound: 1 MHz, 0.75 W cm^−2^; 5 min/day in week 1, then 30 min/day in week 2	Ultrasound 40 kHz, 0.75 W/cm^2^ 30 min × 3×/wk × 4 wk	Ultrasound 2 MHz, 15 W 60 s/day × 7 days	Ultrasound 1 MHz, 1 W/cm^2^ 50% duty cycle 14 days daily	Externally supplied low-intensity pulsed ultrasound (LIPUS)
**Electrical Output**	∼12 V/ ∼36 μA (post-friction correction)	Suboptimal without US (cell traction only); ES enhanced by LIPUS	200 mVpp (transcutaneous, in vivo target)	∼1.69 V (open-circuit, US)	161.2 mV(Vpp)/ 58.7 nA(Ipp); (RMS: 46.7 mV, 18.4 nA)	0.036 μA (peak-to-peak current)	∼130 mV ∼10–70 mV/mm (electric field)	Piezoelectric SF layer; material coefficient and local field are distinct from loaded tissue dose
**Nerve Defect** **Model**	Rat sciatic nerve 15 mm defect 12 weeks	Rat sciatic nerve 15 mm defect 12 weeks	Rat sciatic nerve 15 mm defect 12 weeks	Rat sciatic nerve 10 mm defect 8 weeks	Rat sciatic nerve 8 mm defect 12 weeks	In vitro only RAW264.7 macrophages + BMSCs	Rat sciatic nerve 10 mm defect 12 weeks	10-mm sciatic nerve defect in adult male Sprague–Dawley rats
**Key outcomes (in vivo unless stated)**	CMAP: 19.3 mV vs 20.2 mV (n.s.) SFI: −41.2 vs −42.4 (n.s.) Myelinated density: 1.43 × 10^4^/mm^2^	SFI, CMAP, gastrocnemius fiber area (n.s.) SC proliferation & NGF/BDNF/CNTF: highest in PCL/PVDF-LIPUS group	Reconnection: 4/4 treated vs 1/4 static; SFI improved relative to static controls; small sample, not a clinical success rate	S-100β & NF200 ≈ autograft Neurite length & diff. rate: highest in NGF-BPN US(+) SFI & muscle fiber ≈ autograft	Myelinated axon count: 615 vs 627.8 (n.s.) TO-phase ankle & contracture angle vs autograft (n.s.)	M2 polarization: CD206^+^ 91.02% vs 78.15% (controls) Tuj1 & MAP2 upregulated Ca^2+^-CaMKII-NF-κB suppressed	Axon regen. length: 7.53 ± 0.18 mm (4 wk) SFI wks 9 & 12 vs autograft (n.s.) Myelinated density, myelin thickness, g-ratio ≈ autograft	Increased M2 polarization, GAP43/NCAM1/NF200, enhanced axonal regenreation and remyelination, angiogenesis
**Distinguishing** **Feature**	Fully biodegradable; dual function: ES delivery + real-time nerve repair monitoring via EMG	Trimodal synergy: nanotopography + piezo ES + LIPUS; exogenous US essential for optimal output	Shockwave activation with PS-OCT monitoring; ∼200 mV peak-to-peak is a study output, not a universal optimal dose	Bilayer NGC: piezo ES + thermoresponsive on-demand NGF release by same US stimulus	PFAS-free, biodegradable; fascicle-mimicking 4-channel design; FEA-confirmed axial E-field	MXene-enhanced charge transfer; immune microenvironment modulation (M1→M2)	Record d_33_^eff^ = 43.1 pC N^−1^ (g_33_ = 1909.2 mV m N^−1^); fully biodegradable; Cav3.2 VGCC mechanism	Band-aid-inspired, suture-free, Janus piezoelectric membrane combining tissue adhesion + directional axonal guidance + externally activated piezoelectric stimulation + antibacterial activity + immunomodulation

US, ultrasound; LIPUS, low-intensity pulsed US; ES, electrical stimulation; NGC, nerve guidance conduit; n.s., not significant vs autograft (not proof of equivalence or noninferiority); SFI, sciatic functional index; CMAP, compound muscle action potential.

### 2.1 Self-electrified galvanic devices

Implantable bioelectronic systems have advanced considerably in recent years, particularly in approaches that operate within the physiological environment. One important category is self-electrified galvanic devices, which generate sustained electrical output through spontaneous electrochemical reactions between dissimilar biodegradable metal electrodes [20,21]. The operation of these devices relies on the galvanic effect, in which two dissimilar metals with different electrochemical potentials are coupled in an electrolyte, forming a miniaturized transient galvanic cell. At the anode (e.g., Mg, Zn), oxidation releases electrons and metal ions into the surrounding physiological fluid; at the cathode (e.g., Fe, Mn alloy, Mo), reduction consumes those electrons through reactions with dissolved oxygen and water. This oxidation–reduction potential difference between the two electrodes drives a continuous flow of electrons through the external circuit and ions through the electrolyte, thereby generating a sustained DC electrical output [35]. Unlike nanogenerators, galvanic devices do not require external mechanical or optical activation; instead, they autonomously produce DC electrical stimulation immediately after implantation and continue to operate as long as sufficient reactants, electrolyte access, and electrical continuity are maintained, offering a simple and reliable strategy for sustained postoperative neuromodulation [52]. A representative application of this strategy is bioresorbable Si-Mg galvanic scaffolds, also developed as a symbiotic electrical stimulation scaffold (SESS), which enables long-duration, material-dependent electrical stimulation to enhance nerve regeneration. Overall, this unique architecture, integrating Mo electrodes with a Si semiconductor, ensures stable electrical output, optimizes the electrode-tissue interface, aids in ROS scavenging, and reduces tissue inflammation, offering a promising preclinical therapeutic platform [45].

#### **2.1.1** **Materials selection for** self-electrified galvanic devices

Material selection plays a central role in optimizing galvanic bioelectronic devices, particularly in the design of anodes, cathodes, electrolytes, and packaging materials. In biodegradable galvanic systems, metals with sufficiently separated electrochemical potentials are selected to establish a stable redox couple while maintaining biocompatibility and predictable bioresorption profiles [53]. For instance, Mg is frequently employed as the sacrificial anode because of its highly negative electrochemical potential, favorable biodegradability, and demonstrated compatibility with neural tissues, whereas FeMn alloys or Mo can function as cathodic materials due to their slower corrosion rates and stable electrochemical behavior in physiological electrolytes [54,55]. Appropriate electrode pairing, therefore, requires a comprehensive understanding of the electrochemical characteristics of candidate materials to ensure optimal device performance. Integration of theoretical modeling with advanced material-processing techniques further enables the development of electrodes with high electrical conductivity, robust mechanical integrity, and excellent electrochemical performance [56]. Although bioresorbable metals such as Mg, Zn, and Fe readily degrade in vivo, their corrosion byproducts, particularly elevated local hydroxide ion (OH^−^) concentrations, may induce tissue alkalization and adversely affect cellular activity [53]. Consequently, both the electrode materials and their degradation products or electrochemical byproducts must be carefully considered. In addition, chronic inflammatory responses and fibrotic capsule formation remain persistent challenges following implantation, highlighting the importance of rigorous evaluation of material biocompatibility to minimize long-term inflammatory reactions.

#### 2.1.2 Device architecture for galvanic devices

Beyond material selection, the performance of galvanic bioelectronic systems also depends strongly on device architecture, including electrode geometry, spatial configuration, and encapsulation strategies [35,54,55]. The galvanic cell approach therefore requires careful engineering of these structural parameters to precisely control output voltage, operational lifetime, and degradation kinetics. Electrode thickness, surface area, and spatial arrangement directly influence electrochemical output and stimulation duration [41]. Encapsulation materials and device packaging further regulate electrolyte exposure, ion transport, and mechanical stability while maintaining electrical insulation and structural integrity. These parameters should be specified against the intended treatment period and tested under physiological loading.

Despite their simplicity and ability to provide continuous electrical stimulation independent of patient movement, self-electrified galvanic devices also present several inherent limitations. The electrical output of these systems is largely determined by the electrochemical properties of the electrode materials and cannot be dynamically adjusted after implantation, thereby limiting their adaptability across different stages of nerve regeneration. In addition, uncontrolled degradation kinetics may lead to fluctuations in electrical output or premature loss of functionality, particularly in complex physiological environments. The accumulation of degradation byproducts, such as hydroxide ions or gas evolution, may also alter the local microenvironment and potentially affect cellular responses [56]. Furthermore, integration of aligned fibers with galvanic cells must balance structural guidance, corrosion control, and electrical delivery. Addressing these challenges will require the development of tunable electrochemical systems, advanced encapsulation strategies, and hybrid platforms that combine continuous stimulation with responsive or feedback-controlled functionalities.

### 2.2 Triboelectric nanogenerators

Among the self-powered bioelectronic strategies developed for nerve repair, TENGs have emerged as a prominent platform for converting biomechanical motion into therapeutic electrical energy [[57], [58], [59], [60]]. TENGs operate through the synergistic coupling of contact electrification and electrostatic induction between two dissimilar materials. Contact electrification occurs when two materials with different triboelectric polarities come into contact, causing charge transfer and the formation of surface tribocharges; subsequent separation or relative motion then drives electrostatic induction, redistributing charge between electrodes and generating an alternating potential difference. This coupled process produces high-voltage, low-current alternating electrical outputs during repeated contact–separation cycles, driving current through an external circuit. By utilizing triboelectrification between materials with different electron affinities, mechanical movements such as respiration, limb motion, or tissue deformation can be effectively translated into useable electrical energy [60,61]. The performance and reliability of TENG devices depend strongly on the selection of appropriate materials, making material design a critical aspect of device development. In particular, the materials employed in TENG systems for nerve regeneration must simultaneously satisfy requirements for triboelectric performance, mechanical flexibility, and biocompatibility to enable safe and efficient operation in implantable biomedical environments [62].

#### 2.2.1 Materials for triboelectric layers

TENG design for nerve repair requires the selection of triboelectric material pairs capable of maximizing charge transfer efficiency while maintaining biodegradability and biocompatibility [61,63]. Materials used for triboelectric layer fabrication can generally be classified as organic or inorganic. For example, polymers such as PLGA, (polycaprolactone (PCL), silk fibroin, and cellulose derivatives can be paired to achieve useful charge transfer; the relative triboelectric polarity depends on the counter-material and surface conditions. Materials used for triboelectric layer fabrication can generally be classified as organic or inorganic [61,63,64]. Among organic materials, polydimethylsiloxane (PDMS) is widely employed as a TENG substrate due to its biocompatibility, mechanical flexibility, and ease of fabrication, making it well-suited for implantable bioelectronic applications [[64], [65], [66]]. Fluorinated polymers such as polytetrafluoroethylene (PTFE) and fluorinated ethylene propylene (FEP), commonly referred to as Teflon, exhibit strong negative triboelectric polarity and are therefore highly effective for charge generation when paired with appropriate tribo-positive materials. For biodegradable implantable systems, polymers including PLGA, PCL, PLA, PGA, and silk fibroin are frequently explored as tribopositive layers, while electronegative cellulose derivatives serve as complementary counter-materials, enabling transient TENG-based soft bioelectronic systems [64,67]. In addition, hydrogels, which are water-absorbent polymer networks with mechanical properties similar to biological tissues, provide a compliant, tissue-mimetic interface that is advantageous for soft implantable electronics [68]. Natural materials and other biopolymers have also been used as triboelectric layers because of their triboelectric and dielectric properties. The modification of polymers such as chitosan and cellulose could enhance their triboelectric properties, thereby aiding the design of a novel material with favorable properties [69]. Inorganic materials are primarily used to enhance electrical conduction and triboelectric performance. Metal foils such as Al, Au, and Cu commonly serve as electrodes to facilitate charge collection and transport, providing the stability required for implantable devices. While traditional Au electrodes offer excellent conductivity and stability, biodegradable Mg alloys are particularly attractive for transient implantable TENGs because they combine good electrical conductivity with in vivo degradability, allowing the device to dissolve after completing its therapeutic function [57]. Furthermore, inorganic nanostructures such as zinc oxide, copper oxide, and silver nanoparticles are often incorporated into flexible substrates to increase effective surface area and enhance triboelectric charge generation, thereby improving overall device performance [70,71]. Importantly, polymers such as PDMS, PTFE, and FEP, despite their favorable biocompatibility and chemical stability, remain non-resorbable in physiological conditions. As a result, they are incorporated into a biodegradable scaffold, such as lipids, proteins, and polysaccharides, to minimize the residual material burden. The biological fate of implantable bioelectronics should therefore be evaluated at the device level rather than the material level, considering the degradation, clearance, and long-term tissue response of all implanted components, including substrates, electrodes, conductors, encapsulation layers, adhesives, and interconnects [72,73].

#### 2.2.2 Device archictecture for TENGs

Most TENG systems developed for neural repair adopt the vertical contact-separation (CS), single-electrode (SE) mode, freestanding (FS) mode, and single-electrode (SE) mode, which are widely favored because of their structural simplicity, reliable electrical output, and ease of encapsulation for implantable or wearable biomedical applications [[64], [65], [66],^71^]. In the CS mode, periodic vertical motion brings two dielectric layers into contact and separation, enabling efficient charge transfer and high electrical output through repeated triboelectric interactions. By contrast, the SE mode simplifies the device architecture by employing a single electrode referenced to ground, allowing a freely moving object to serve as the counter-triboelectric layer (Fig. 2) [74].

**Fig. 2:**
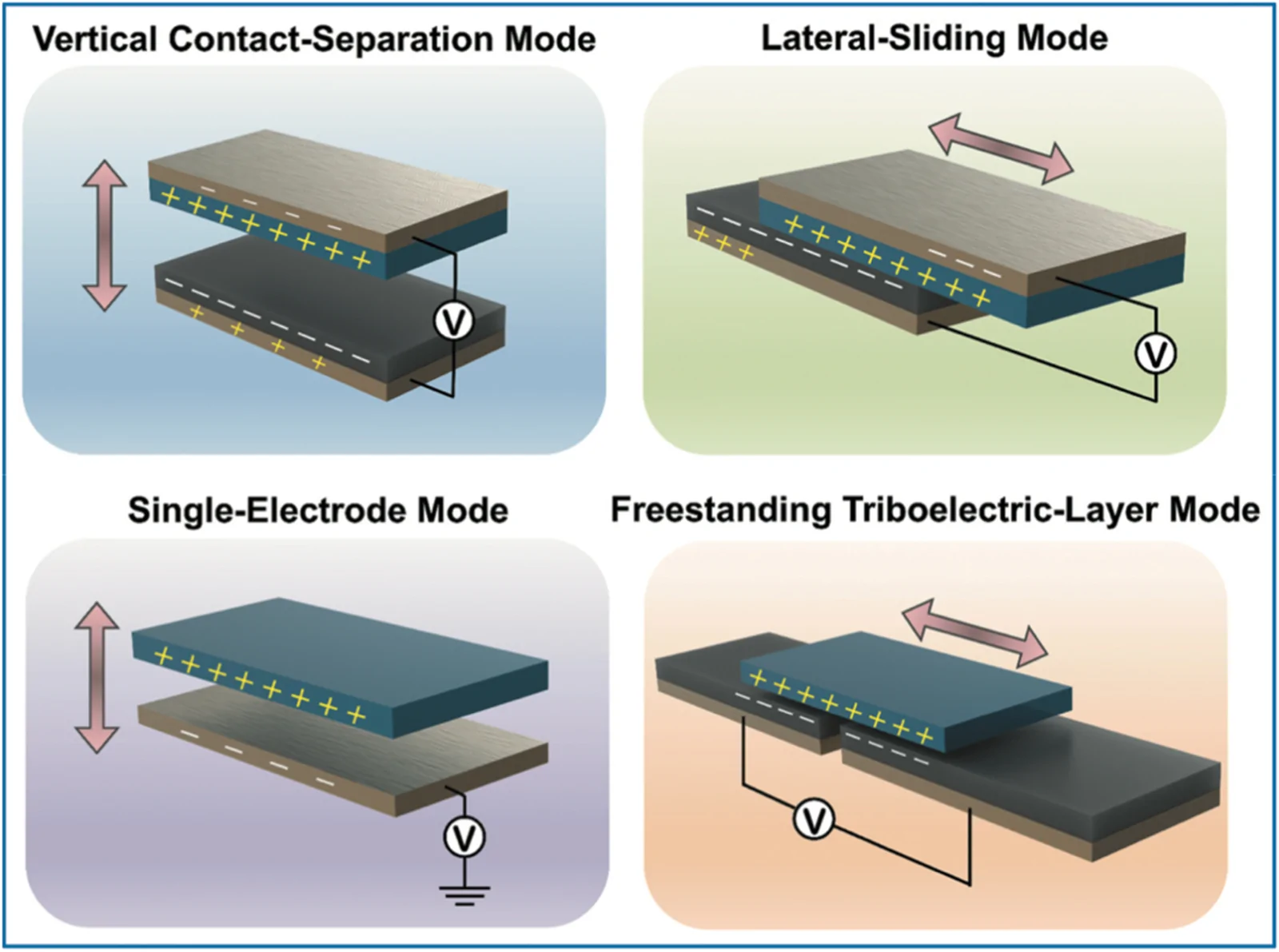
Four fundamental modes of TENG (Reproduced with permission [69]. Copyright © 2022, Taylor & Francis). Schematics are not drawn to scale; dimensions in microscopy panels refer to the original panel scale bars, not the displayed schematic size.

The operational mode of independent autonomous (freestanding) friction triboelectric-layer mode TENG consists of two fixed electrodes and a freely moving triboelectric layer that generates AC through periodic charge induction and electron transfer between the electrodes. It can operate between stationary electrodes, reducing material wear, lowering energy loss, and extending device lifespan. This mode effectively generates stable, self-powered electrical stimulation [75]. To maximize electrical performance, device interfaces are often engineered with micro- or nanostructured surfaces that increase effective contact area and improve triboelectric charge generation. In addition to harvesting biomechanical vibrations from physiological motion, external low-intensity ultrasound has emerged as an effective wireless actuation strategy to drive contact–separation in deeply implanted TENGs [76]. By converting transcutaneous acoustic energy into alternating electrical pulses, ultrasound-driven TENGs provide on-demand power supply and synchronized bioelectric stimulation for neural repair without invasive tethers. Furthermore, targeted structural design and biocompatible encapsulation are crucial for stabilizing the electrode–tissue interface, thereby ensuring predictable resorbability and reliable therapeutic output throughout the nerve regeneration window [66].

Several device configurations have been explored to adapt TENG technology for neural regeneration. Implantable TENG systems can be positioned adjacent to injured nerves, where subtle physiological movements are converted into localized electrical stimulation directly delivered to the regeneration site [18,[23], [24], [25], [26],^52^]. In addition, TENG-integrated conductive nerve conduits have been developed to simultaneously bridge peripheral nerve gaps while delivering self-powered electrical stimulation along the regeneration pathway [41,64,67]. Through these design strategies, TENG-based systems provide a promising platform for activity-dependent neuromodulation when physiological motion supplies sufficient energy. For instance, a self-powered electrical stimulation platform integrating a TENG with conductive poly(3,4-ethylenedioxythiophene) (PEDOT)-reduced graphene oxide (rGO) hybrid microfibers has demonstrated significant potential for neural tissue engineering. The PEDOT-rGO scaffold provides excellent electrical conductivity and cytocompatibility, promoting mesenchymal stem cell (MSC) adhesion, proliferation, and neural lineage commitment. The conductive hydrogel scaffold coupled with pulsed electrical stimulation generated by a TENG and powered by biomechanical energy from human walking markedly enhanced MSC neural differentiation without the need for additional biochemical induction factors [34,77]. This strategy highlights the potential of wearable, self-powered bioelectronic systems to provide motion-dependent electrical stimulation, offering a promising approach for stem cell-based nerve regeneration and the development of next-generation implantable neural scaffolds. Thus, TENGs harness passive human biomechanical energy to generate a self-powered system for ES-based therapeutic interventions [34].

Despite the rapid development of TENG-based neurostimulation systems, several critical challenges remain that may limit their clinical translation. One major limitation is the dependence of electrical output on sufficient biomechanical motion, which can be significantly reduced during the early postoperative phase when patients are immobilized. This mismatch between peak therapeutic demand and minimal physiological activity may lead to suboptimal stimulation during the most critical window for nerve regeneration. In addition, the relatively high-voltage but low-current characteristics of TENG outputs raise questions about the efficiency of charge delivery at the tissue interface, particularly in conductive biological environments where signal attenuation may occur. Furthermore, long-term device stability under repeated mechanical cycling and the reliability of encapsulation strategies in vivo remain insufficiently explored [9,58]. Future efforts should therefore focus on improving energy conversion efficiency during low-amplitude motion, integrating hybrid energy-harvesting mechanisms, and developing adaptive output-regulation strategies to ensure consistent and physiologically relevant stimulation profiles.

### 2.3 Piezoelectric nanogenerators

PENGs represent a promising technology for implantable bioelectronics by enabling the direct conversion of mechanical energy into electrical signals. These devices operate based on the piezoelectric effect, an intrinsic property of certain non-centrosymmetric crystalline materials in which mechanical deformation induces a net electrical dipole. When such a material is subjected to mechanical stress or strain, the displacement of positive and negative charge centers within its crystal lattice creates an internal polarization, generating a potential difference across the material. This strain-induced polarization drives the flow of free charges through an external circuit, producing an alternating electrical output as the mechanical deformation is applied and released [60,[78], [79], [80], [81]]. Through this mechanism, PENGs can harvest biomechanical energy from physiological activities, such as body motion, muscle contraction, or cardiovascular pulsation, or from externally applied stimuli such as low-intensity pulsed ultrasound (LIPUS), converting these inputs into localized therapeutic electric fields [30,31,[47], [49], [80], [81], [82]]. Owing to their small size, high energy-conversion efficiency, and mechanical robustness, PENGs provide a practical platform for developing durable and miniaturized biomedical implants [[83], [84], [85]]. The continued advancement of this technology not only expands the capabilities of implantable bioelectronic systems but also opens new opportunities for innovative therapeutic strategies in modern healthcare.

#### 2.3.1 Materials selection for PENGs

PENGs materials used for nerve repair are designed to convert mechanical energy from physiological motion or external stimuli, such as ultrasound, into localized electrical stimulation that supports neural regeneration. These materials enable the development of self-powered, wireless nerve-guiding conduits that enhance Schwann cell migration, promote axonal growth, and accelerate peripheral nerve repair, with activation requirements determined by the energy source. Common piezoelectric materials include polymers, ceramics, and composite systems [[85], [86], [87]]. Among them, PVDF and its copolymers (e.g., PVDF-TrFE) are widely used due to their flexibility, biocompatibility, and ability to generate electrical signals under mechanical deformation [88,89]. To overcome the high intrinsic impedance of PVDF and optimize charge collection, PVDF is frequently integrated with conductive polymers such as PEDOT. The incorporation of PEDOT forms a complementary conductive network that significantly enhances electrical signal transmission across the piezoelectric matrix. Additionally, Zinc oxide (ZnO) nanostructures are also frequently incorporated because of their strong piezoelectric coupling and favorable biocompatibility [90,91]. In addition, composite materials that combine piezoelectric components with natural polymers such as collagen can improve both electrical performance and biological integration [[85], [86], [87],^92^,^93^]. Moreover, electroactive microfibrous scaffolds generated using silk fibroin and PEDOT: PSS (polystyrene sulfonate) promote peripheral neural tissue engineering by guiding axons across nerve gaps and connecting with distal injured nerve via biological signal transduction [6].

Traditional piezoceramics such as lead zirconate titanate (PZT) exhibit excellent piezoelectric properties and high energy conversion efficiency; however, the presence of lead raises concerns regarding biocompatibility and environmental safety [[94], [95], [96]]. Consequently, lead-free piezoelectric alternatives, including barium titanate (BaTiO_3_), potassium–sodium niobate (KNN), and sodium bismuth titanate (NBT), have been increasingly explored for implantable bioelectronics. However, a lead-free composition alone does not establish complete biodegradability or biological clearance, as many ceramic particles remain persistent under physiological conditions. Likewise, biodegradable piezoelectric polymers such as poly(L-lactic acid) (PLLA), PGA, and polyhydroxyalkanoates (PHA) are also attractive candidates because they can generate electrical signals under mechanical stress, which is gradually eliminated, making them favorable for systemic clearance [92,97,98]. In practical PENG devices, these materials are integrated with biocompatible substrates, such as silicon or flexible polymers (e.g., polyimide), which provide structural support and conformability to biological tissues [99,100]. Electrode materials, typically noble metals such as Au or Pt, are used to collect and transport the generated electrical charge, while biocompatible encapsulation layers, including PDMS, PDVF, polyurethane, and medical-grade silicone, protect the device and ensure long-term stability and safety in implantable environments [[101], [102], [103]]. These metal-based bioelectronic devices, although they provide excellent electrical performance and long-term stability, are non-resorbable in nature. Therefore, the biological fate of implantable PENGs should be evaluated at the whole-device level, considering the degradation, retention and long-term tissue response of all implanted components rather than the piezoelectric polymer alone.

#### 2.3.2 Device architecture for PENGs

PENGs for nerve repair are flexible, biocompatible bioelectronic scaffolds that convert mechanical energy from body motion or external stimuli into electrical stimulation that promotes neural regeneration [30,31,44,80,81,82,47]. These devices are often engineered as nerve-guiding conduits that provide structural support across nerve defects while generating bioelectric signals that mimic endogenous electrophysiological activity, thereby enhancing Schwann cell proliferation and axonal growth [30,31,37,47]. Beyond macro-scale body motion, localized piezo-potential can also be generated at the cellular level by cell traction forces, creating micro-electric fields ranging from a few millivolts to several tens of millivolts (comparable to physiological membrane potentials). As battery-free and lead-free platforms, piezoelectric materials deliver localized electrostimulation that effectively promotes cell migration, calcium influx, and proliferation. Specifically, as crawling cells exert traction forces on the piezoelectric fibers, the resulting mechanical deformation generates self-stimulating electrical cues, creating a proposed local electromechanical interaction; this does not establish a measured therapeutic dose or a validated closed-loop controller.

In device design, material selection is crucial, with piezoelectric polymers such as polyvinylidene fluoride (PVDF), PLLA, and PVDF-co-hexafluoropropylene (PVDF-HFP) widely used for their flexibility and ability to generate electrical charge under mechanical deformation. Electrospinning is commonly employed to fabricate aligned nanofibrous structures that resemble the extracellular matrix and facilitate cell adhesion and axonal guidance [37,47,104]. Structurally, PENG systems are typically designed as nerve-guiding conduits that bridge nerve gaps, sometimes incorporating conductive nanomaterials to enhance electrical output. Mechanical stimulation from physiological movement or ultrasound induces localized electrical signals that activate Schwann cells and accelerate axonal outgrowth. These systems offer several advantages, including self-powered and wireless operation, enabling in situ electrical stimulation when the required mechanical input is available. In addition, many piezoelectric polymers, including PLLA, are biodegradable, allowing the implant to degrade gradually after nerve regeneration and potentially avoiding the need for removal once all implanted components have cleared [92,97,98,105]. To further advance these bioelectronic interfaces, smart manufacturing strategies such as 3D and 4D bioprinting have been integrated with ultrasound-modulated piezotechnology to enable non-invasive, personalized treatments [106]. For instance, combining 3D-bioprinted hydrogel scaffolds with wireless ultrasound activation provides a versatile strategy to enhance neural stem cell differentiation and functional peripheral nerve regeneration in vivo. Furthermore, 4D-printed bioelectronic interfaces utilizing dynamic, self-healing materials, such as poly(urethane urea amide) (sPUUA) elastomers, pave the way for shape-adaptive neural scaffolds [9]. Thanks to dynamic thiocarbamate chemistry and disulfide bond formation, these 4D-printed scaffolds possess intrinsic shape-memory and self-healing capabilities, allowing them to dynamically adjust to the shape and dimensions of target nerve tissues. This significantly improves the electrode-nerve interfacial conformity, accommodates small peripheral nerves, and optimizes signal transmission efficiency.

Although piezoelectric nanogenerators have demonstrated significant promise for self-powered nerve regeneration, their performance remains strongly dependent on the availability and magnitude of mechanical stimulation. In many clinical scenarios, particularly in cases of severe injury or immobilization, endogenous mechanical inputs may be insufficient to generate therapeutically effective electrical outputs. While ultrasound-activated systems offer a compelling solution, they introduce additional complexity in terms of external equipment, energy delivery efficiency, and potential safety concerns associated with prolonged acoustic exposure. Moreover, the trade-off among mechanical flexibility, piezoelectric performance, and biodegradability remains a key materials challenge, as high-performance piezoelectric materials often lack optimal biocompatibility or degradation profiles [16]. Future research should therefore prioritize the development of high-sensitivity piezoelectric materials capable of operating under ultra-low mechanical stimuli, as well as the integration of wireless and minimally invasive activation strategies to expand their applicability across diverse clinical conditions.

### 2.4 Stimulation dose and electrochemical safety

Therapeutic efficacy of biodegradable bioelectronics cannot be determined by generator voltage alone, as electrical exposure depends on current density, charge density, waveform, pulse width, frequency, electrode geometry, and stimulation duration. It also depends on material stability and degradation behavior. Galvanic devices may induce local pH changes, gas evolution, and excessive ion release during electrode corrosion, whereas TENGs and PENGs require assessment of leakage current, wet-state output degradation, rectification efficiency, acoustic heating, and mechanically induced tissue injury [41,42,44]. Quantitative parameters should be reported under physiologically relevant loads; for example, Mg–FeMn galvanic systems generated electric fields of 20–250 mV mm^−1^, while ACT-TENGs exhibited 7.8 V output voltage at 40 MΩ load and 60 μA current at 1 Ω load. Ultrasound-driven APNF-NGC piezoelectric interfaces produced 161.2 mV peak-to-peak voltage and 58.7 nA peak-to-peak current at 40 kHz excitation. Hereafter, these values represent device outputs rather than direct tissue-level stimulation doses and require validation through loaded measurements, tissue-equivalent models, and in vivo interface characterization. The stimulation parameters reported in in vitro and in vivo studies represent platform-specific preclinical conditions; however, relevant voltage outputs (power, stimulation lifetime, acoustic carrier, waveform) are specifically bioelectronic device outputs. Translation towards clinical applications requires validation under relevant loading conditions, large-animal validation, and long-term in vivo evaluation. Further, electrochemical safety assessment should include charge accumulation, electrode polarization, leakage current, local pH variation, ion release, gas evolution, thermal effects, and degradation-induced changes in electrical output to establish clinically relevant stimulation windows. Establishing uniform stimulation-dose parameters and electrochemical safety criteria would facilitate improvement of biodegradable bioelectronics and hasten their translation into clinically useable nerve regeneration therapies.

## 3 Proposed mechanism for neuro-regeneration

The strategic convergence of self-electrified galvanic devices and nanogenerators has established a powerful framework for promoting nerve regeneration, supported by partly overlapping cellular responses alongside their respective functional advantages. At the cellular level, electric fields may facilitate repair through galvanotaxis, in which actively generated electric fields guide growth cone migration toward the injury site via asymmetric accumulation of PIP3 and activated Rac1 GTPase on the cathodal side [57,107]. Directional responses in experimental systems do not establish universal polarity requirements or prevent neuroma formation [[107], [108], [109]]. Furthermore, these active electrical cues modulate Schwann cell behavior via the integrin β1/FAK pathway, propelling Schwann cells into a proliferative repair phenotype that accelerates myelin debris clearance and enhances secretion of neurotrophic factors including NGF, BDNF, and GDNF [20,110,111]. Beyond the neural cells themselves, selected platforms influence the local immune microenvironment, including changes in macrophage polarization from the pro-inflammatory M1 toward the pro-regenerative M2 phenotype, releasing anti-inflammatory cytokines such as IL-10 and TGF-β to hasten the transition from Wallerian degeneration to active regeneration [112,113].

Despite these overlapping responses, self-electrified galvanic cell devices and nanogenerators differ fundamentally in their energy transduction principles, output waveform characteristics, and structural integration. Galvanic cell platforms harness spontaneous electrochemical redox reactions between dissimilar metal electrodes, using interstitial body fluids as the electrolyte to generate a continuous, unidirectional DC electric field (20–250 mV mm^−1^) that is entirely independent of the patient's physical activity [20,22,45]. Movement-independent DC output may be advantageous during the acute postoperative window when patients are immobile, as it delivers sustained stimulation precisely when early neurotrophic responses are most critical. In contrast, nanogenerators transduce mechanical energy derived from physiological motion or external ultrasound into pulsed electrical signals (AC output) [18,24] [99, 100, 101]. These motion-synchronized pulses have been broadly associated with downstream signalling responses, including GAP43 upregulation, RAS/MAPK and PI3K-AKT pathway activation, and in select platforms, macrophage M1 to M2 polarization via suppression of the Ca^2+^/CaMKII-NF-κB axis [28,49].

Additionally, immune-regulated pathways, like focal adhesion kinase (FAK) mediated mechanotransduction, regulate cell adhesion, migration, cytoskeleton reorganization, whereas (Yes-associated protein) YAP/ (transcriptional coactivator with PDZ-binding motif) TAZ, signaling pathway shows mechanical stimuli with transcriptional responses to coordinate nerve regeneration. Electrical stimulation should be distinguished from the effects of scaffold architecture and external mechanical stimulation. Fibrous nerve guidance conduits, whether used in galvanic or PENG platforms, provide topographical guidance that promotes Scaffold architecture for Schwann cell migration and directional axonal elongation independently of electrical stimulation. In contrast, the therapeutic electrical cues are generated through distinct energy-transduction mechanisms. For example, PENG-based conduits (e.g., PCL–β-glycine nanocomposites) generate electrical stimulation through piezoelectric polarization induced by mechanical deformation, whereas implantable Zn–O galvanic cells produce continuous electrical stimulation via electrochemical redox reactions between biodegradable metal electrodes [22,114]. External mechanical inputs, such as ultrasound or tissue deformation, activate PENGs but may also independently influence mechanotransduction pathways; therefore, mechanically stimulated controls without electrical output are essential to isolate the specific contribution of electrical stimulation. Furthermore, galvanic devices produce a fixed electrical output determined by electrode chemistry, whereas PENG output can be tuned through material composition, device architecture, and mechanical loading, with ultrasound-driven systems enabling externally programmable stimulation. Macrophage polarization and the inflammasome signaling pathway influence the formation of a pro-regenerative microenvironment, thereby recruiting bioelectrical cues using external or self-powered bioelectronics as a modulator of cellular communication, tissue regeneration, and functional recovery [52,115].

## 4 Platform technologies of self-powered bioelectronics

The development of self-powered bioelectronics has enabled the integration of electrical stimulation directly into therapeutic platforms for peripheral nerve regeneration. Rather than relying solely on externally powered stimulators, these systems generate or sustain electrical cues through distinct energy-transduction strategies. In this review, three representative platforms are considered: self-electrified galvanic devices, TENGs, and PENGs. Although all three approaches aim to provide localized electrical stimulation, their energy sources, output characteristics, and dependence on physiological or external activation are fundamentally different. Galvanic devices generate continuous electrical stimulation through electrochemical reactions and can therefore operate independently of patient movement, whereas TENGs and PENGs convert mechanical deformation or externally supplied mechanical energy into electrical signals. These differences directly influence where and when each platform can be applied during nerve repair. Accordingly, the following sections focus on how these technologies have been implemented in nerve guidance conduits, implantable interfaces, and other therapeutic configurations, with emphasis on their demonstrated regenerative outcomes. Particular attention is given to the relationship between device architecture, stimulation characteristics, degradation behavior, and biological response. Rather than comparing platforms solely on electrical output, their applications are considered in the context of the specific therapeutic requirements and experimental conditions under which regeneration was evaluated.

### 4.1 Self-electrified galvanic devices

The concept of bioelectric stimulation dates back to Luigi Galvani's discovery of bioelectricity in the frog sciatic nerve, which established the fundamental role of electrical signals in physiological processes such as nerve conduction, cardiac rhythms, and wound healing [114]. As outlined in Section 2.1, galvanic devices provide movement-independent DC stimulation. For the nerve-repair applications below, the key design variables are operational lifetime, electrode–tissue exposure, and integration with the conduit, rather than nominal power alone (Section 2.4). In the field of galvanic-cell-based self-powered bioelectronics, Lan Yin and colleagues have pioneered the integration of miniaturized bioresorbable galvanic cells into nerve guidance conduits and neural interfaces for autonomous electrical stimulation. By utilizing interstitial body fluids as electrolytes, these devices generate localized direct-current electric fields without external power supplies or percutaneous leads (Table 2). Such self-powered platforms provide sustained electrostimulation during critical postoperative recovery periods, thereby promoting axonal regeneration and Schwann cell proliferation. Importantly, bioresorbable designs may avoid secondary removal, while combined stimulation and recording provide a promising “monitor-and-treat” approach that still requires clinical validation. A representative application of this strategy is bioreabsorbable Si-Mg galvanic scaffolds, also developed as a SESS, which enables long-duration, material-dependent electrical stimulation to enhance nerve regeneration. Overall, this unique architecture, integrating Mo electrodes with a Si semiconductor, ensures stable electrical output, optimizes the electrode-tissue interface, aids in ROS scavenging, and reduces tissue inflammation, offering a promising preclinical therapeutic platform [45].

The initial proof of concept was reported in 2020, when Yin's group developed a fully biodegradable and self-electrified nerve conduit incorporating an Mg–FeMn galvanic cell [20]**.** The device consisted of a 3.5 μm magnesium anode and a 1.5 μm iron–manganese cathode, capable of generating stable electric fields of 20–250 mV/ mm using physiological fluids as the electrolyte (Fig. 3a). This autonomous electrical stimulation significantly enhanced calcium activity and neurite outgrowth in dorsal root ganglion neurons (P < 0.01), while simultaneously accelerating Schwann cell proliferation and upregulating neurotrophic factors including NGF, BDNF, and VEGF. In a 10-mm rat sciatic nerve defect model, the electroactive conduit achieved a sciatic functional index (SFI) of (41.51 ± 5.47) at 12 weeks, approaching the functional recovery observed with autografts and markedly outperforming hollow conduit controls (69.82 ± 3.06). Improvements in compound muscle action potentials and reduced muscle atrophy further demonstrated the therapeutic potential of this self-powered electroceutical platform for nerve regeneration.

**Fig. 3:**
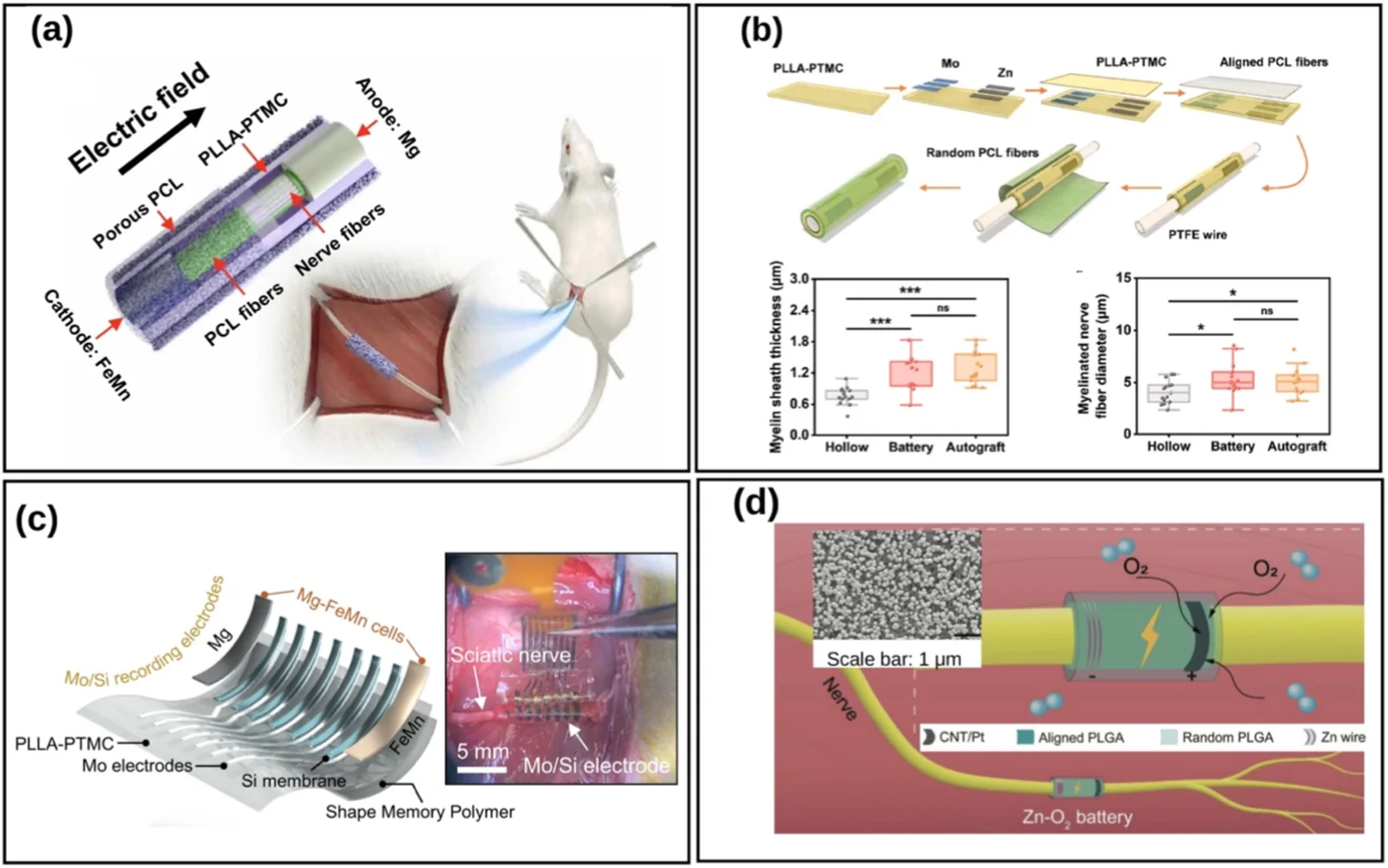
a) Schematic of a biodegradable self-electrified NGC for sciatic nerve regeneration, comprising a porous PCL outer layer, PLLA-PTMC film, Mg-FeMn galvanic cell, and inner electrospun PCL fibers (Reproduced with permission [20]. Copyright © 2020, AAAS). b) Fabrication schematic of the self-powered NGC, consisting of an aligned PCL guidance film, a PLLA-PTMC-encapsulated Zn-Mo battery, a PLLA-PTMC substrate, and a randomly oriented PCL support film. Average of myelin sheath thickness and myelinated fiber diameters (Reproduced with permission [21]. Copyright © 2024, ELSEVIER). c) Schematic of the biodegradable neural interface, comprising a PLLA-PTMC substrate, Mg-FeMn galvanic cell, and Mo/Si recording electrode array implanted on the sciatic nerve (Reproduced with permission) [9]. Copyright © 2025, Springer Nature). d) Schematic of a tubular Zn-O_2_ battery wrapped around an injured nerve as an in vivo power source, with SEM images of the CNT/Pt cathode (Reproduced with permission [22]. Copyright © 2023, John Wiley and Sons). Schematics are not drawn to scale; dimensions in microscopy panels refer to the original panel scale bars, not the displayed schematic size.

Subsequent work from the same group addressed key limitations of magnesium-based systems, namely rapid degradation and hydrogen evolution, through the development of a zinc–molybdenum (Zn–Mo) battery–powered nerve conduit (Fig. 3b) in 2024 [21]. By integrating aligned PCL fibers with a dissolvable Zn–Mo microbattery, the device provided both topographical guidance and sustained electrical stimulation, extending the effective stimulation window by approximately sixfold relative to earlier Mg-based designs. The battery, composed of a sintered Zn particle anode and a Mo particle cathode, produced a stable output voltage of ∼0.5 V for up to 50 h. In vivo evaluation in a rat sciatic nerve injury model demonstrated accelerated axonal extension (8.43 ± 0.44 mm at 3 weeks) and enhanced Schwann cell metabolic activity. Long-term outcomes at 12 weeks revealed significantly larger nerve fiber diameters (5.33 ± 1.55 μm, p < 0.05) and thicker myelin sheaths (1.30 ± 0.30 μm, p < 0.001), with functional recovery approaching that achieved with autografts. These results highlight the advantages of zinc-based bioresorbable electronics for sustained electrotherapeutic stimulation.

Building upon these advances, Wang et al. from Yin's group further expanded the concept by introducing Bio-Restor, a multifunctional bioresorbable neural interface that integrates both therapeutic stimulation and neural monitoring [9]. The system retains the Mg–FeMn galvanic cell architecture to deliver localized electric fields (20-250 mV mm^−1^) that accelerate axonal regeneration by promoting Wallerian degeneration and upregulating regeneration-associated genes (Fig. 3c). In a 10-mm rat sciatic nerve defect model, the device produced significantly higher axon densities than non-stimulating controls by week 3 (p < 0.001) and enabled earlier muscle reinnervation, as evidenced by evoked hindlimb movements. Importantly, Bio-Restor incorporates a 7-channel Mo/Si neural recording array capable of electrophysiological monitoring for 5.5–8.5 weeks, allowing simultaneous therapeutic stimulation and neural signal acquisition. By combining stimulation, monitoring, and AI-assisted diagnostic analysis within a fully bioresorbable platform, this system provides a basis for future closed-loop therapy; simultaneous recording does not show that a controller automatically adjusted stimulation. A recent study by Wang et al., in 2026 developed a fully bioresorbable SESS. The device is based on Si-Mg galvanic cell system integrated into an aligned PCL fibrous scaffold. The SESS utilizes body fluids as an electrolyte to trigger galvanic reactions between Mg and Si, enabling continuous, material-dependent electrical stimulation in vivo. The scaffold demonstrated an impedance of approximately 200 kΩ, a maximum power output of ∼0.15 μW, and an initial voltage output of ∼400 mV, which gradually decreased to ∼80 mV after 3 weeks in vitro and ∼50 mV after 90 days in vivo, providing long-term stimulation during nerve repair. In vitro studies demonstrated excellent cytocompatibility and neuroregenerative potential. Electrical stimulation from SESS enhanced cell migration, promoted directional growth of PC12 neuronal cells, and increased axonal extension from dorsal root ganglia (DRG) explants. SESS significantly improved axonal regeneration and remyelination, producing an axonal diameter of 4.56 ± 1.34 μm and myelin thickness of 0.50 ± 0.12 μm in rat sciatic nerve defect model. Further, transcriptomic analysis revealed that SESS-mediated electrical stimulation activated pathways associated with axon guidance, PI3K–Akt signaling, calcium signaling, extracellular matrix interaction, and cell adhesion, while promoting anti-inflammatory responses through increased expression of Tgfb1, Tgfbr1, and Il 10 and suppression of inflammatory genes [45].

Complementing these developments, Li et al., in 2023 reported an implantable tubular Zn-O_2_ battery (Fig. 3d) designed as a high-performance power source for in situ neural electrical stimulation [22]. The reported electrode volume of 0.86 mm^3^ and energy density of 231.4 mWh cm^−3^ in vivo studies proved that ES promotes regeneration of injured eyes, comparable to clinical gold standards. Its tubular architecture allows direct wrapping around injured nerve segments, enabling localized electrical modulation that significantly enhances the regeneration of 10-mm sciatic nerve defects in rats followed for 12 weeks. The control unit of the tubular Zn-O_2_ implantable battery could be further integrated with the miniaturized device for personalized treatment effects in the future. This strategy addresses a key challenge in implantable bioelectronics, namely the development of compact yet high-energy power sources, and provides an important technological foundation for future self-powered neural therapeutic systems.

Collectively, these studies illustrate the rapid evolution of galvanic-cell-based bioresorbable electroceuticals, progressing from early proof-of-concept self-powered conduits to multifunctional neural interfaces capable of simultaneous stimulation and monitoring. Continued advances in biodegradable power sources, device architectures, and closed-loop control strategies are expected to further accelerate the translation of self-powered electroceutical systems for clinical nerve repair.

### 4.2 Triboelectric nanogenerators

Triboelectric nanogenerators assist nerve repair by converting biomechanical energy generated from physiological activities, such as muscle movement, heartbeat, and respiration, into electrical stimulation that promotes neural regeneration. Here, the emphasis is on how activation, device lifetime, and model selection affect the interpretation of nerve-repair outcomes [52,78].

The electrical stimulation generated by TENGs facilitates nerve repair through multiple biological pathways, thereby promoting axonal regeneration and accelerating the outgrowth and elongation of axons across injured regions [4]. At the cellular level, stimulation can upregulate regeneration-associated proteins such as GAP-43, supporting neuronal survival and differentiation. Furthermore, stimulation of peripheral nerves helps maintain neuromuscular signaling, thereby reducing muscle atrophy and improving motor recovery [26]. A major advantage of TENG-based systems is their self-powered nature, which eliminates the need for external batteries and enables activity-dependent electrical stimulation driven by natural body movements. Moreover, TENG devices may be integrated into future closed-loop systems, provided that sensing is linked to a validated rule that adjusts the delivered stimulus, thereby enabling adaptive neuromodulation therapies.

An important early milestone was reported by Zheng et al., in 2016, who demonstrated the fully biodegradable triboelectric nanogenerator (BD-TENG) designed to function as a lifetime-tunable power source for transient implantable electronics. The device was fabricated using various bioresorbable polymers, including PLGA, PCL, PHB, silk fibroin, and chitosan, combined with dissolvable metal electrodes such as magnesium, molybdenum, or iron. This configuration enabled controllable electrical outputs, with a PLGA-based BD-TENG producing a peak open-circuit voltage of 40 V and a short-circuit current of 1 μA. The generated stimulation guided the orientation and growth of nerve cells, demonstrating a transient power-source concept rather than repair across an injured peripheral nerve gap. In vivo experiments further showed that degradation after implantation with short-term tissue compatibility; these observations do not establish long-term inflammatory safety for every material combination. By enabling programmable device lifetimes that align with therapeutic timelines, this work established a foundational strategy for transient electroceutical devices that eliminate the need for surgical removal.

Expanding the concept of autonomous stimulation, Jin et al., in 2021 developed a physiologically self-regulated, fully implantable neural electrical stimulation system (FI-NES) designed to enhance the repair of long-segment peripheral nerve injuries [18,24]. To ensure sufficient therapeutic output even under subtle physiological motion, the system integrates a triboelectric–piezoelectric hybrid nanogenerator (TP-hNG, Silicone rubber nano-wrinkle film/PVDF, ZnO nanofibers) with a nanoporous nerve guidance conduit (Fig. 4a). This hybrid architecture produces electrical stimulation that dynamically responds to different physiological states, enabling synchronized stimulation during daily activities. In a 15-mm rat sciatic nerve defect model, the FI-NES platform significantly improved functional recovery and electrophysiological performance, achieving compound muscle action potential amplitudes comparable to those of autograft repair. Histological analyses further confirmed enhanced myelination and improved muscle preservation relative to conventional nerve conduits. This work demonstrated the potential of hybrid energy-harvesting systems to provide adaptive, activity-dependent stimulation for nerve regeneration.

**Fig. 4:**
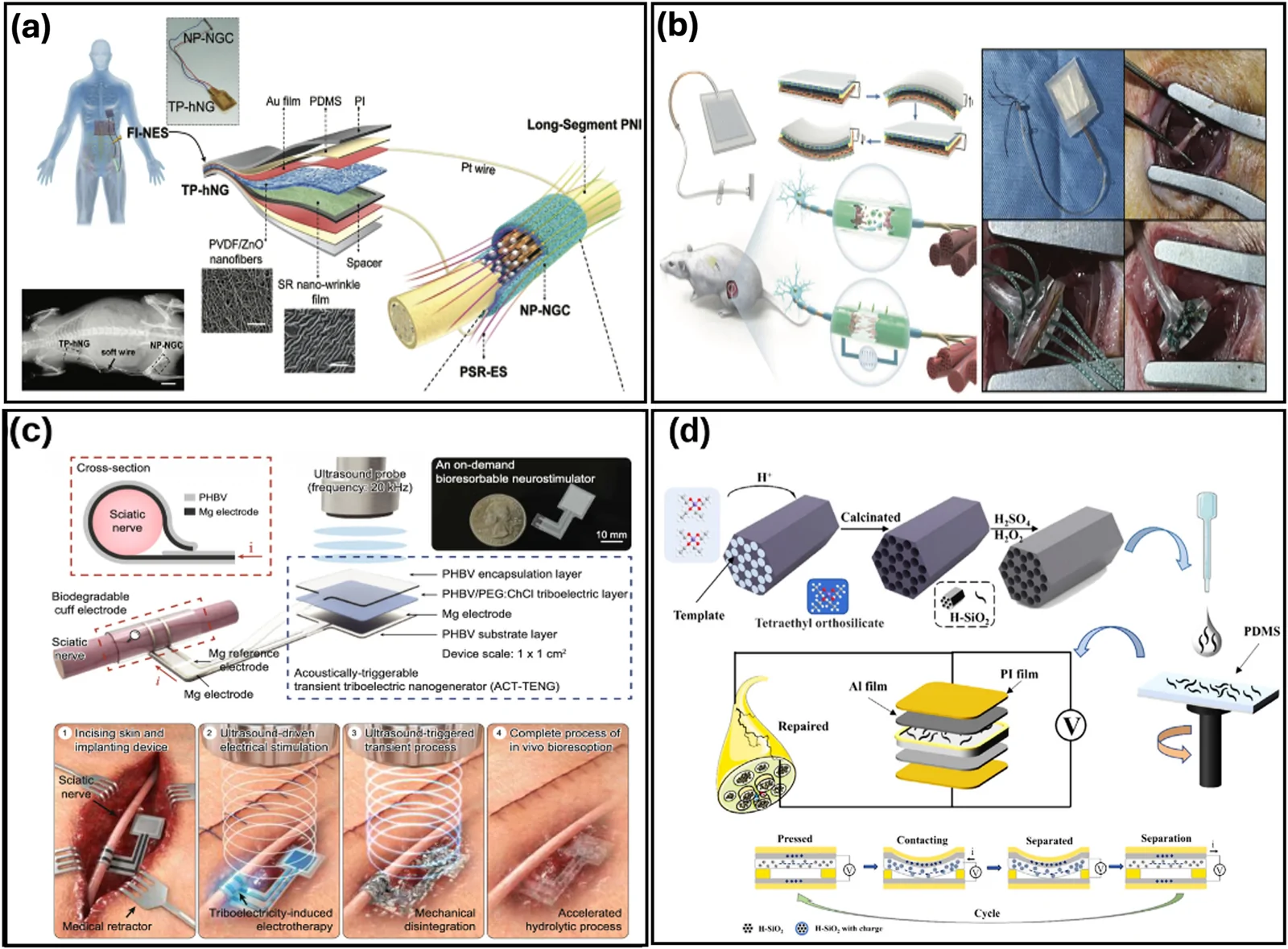
(a) Working principle of the FI-NES system for long-segment peripheral nerve repair, with in vivo X-ray imaging confirming device implantation in a rat model (Reproduced with permission [18]. Copyright © 2021, John Wiley and Sons). (b) Design and working principle of the ISR-NES system, illustrating the energy-harvesting mechanism of the Cs-TENG, along with photographs of the device and its implantation on the injured sciatic nerve (Reproduced with permission [24]. Copyright © 2022, John Wiley and Sons). (c) Overall structure of the ACT-TENG-based neurostimulator with an expanded view of the device components and cuff electrode, and the proposed clinical protocol for peripheral nerve electrotherapy (Reproduced with permission) [25]. Copyright © 2023, Springer Nature).(d) Fabrication schematic of the H-SiO_2_-enhanced TENG and illustration of its electrical output generation principle during contact-separation cycles (Reproduced with permission [26]. Copyright © 2026, ELSEVIER).

Building upon this concept of physiologically synchronized stimulation, Zhou et al. introduced an implantable self-regulated neural electrical stimulation (ISR-NES) system that synchronizes therapeutic pulses with physiological body movements [24]. The system employs a contact–separation triboelectric nanogenerator composed of PDMS and PA6 dielectric layers reinforced with silver nanowires (Fig. 4b), enabling stable in vivo voltage outputs of approximately 2.1–2.2 V over a four-week period. In a rat sciatic nerve injury model, the ISR-NES platform significantly accelerated both motor and sensory functional recovery compared with untreated controls. Electrophysiological and histological analyses further revealed enhanced nerve conduction, increased expression of regeneration-associated protein GAP-43, and reduced denervation-induced muscle atrophy. This motion-driven, battery-free paradigm represents a key step toward physiologically responsive neurostimulation systems that automatically adapt stimulation patterns to the host's natural movements [116]. For instance, a self-powered electrical stimulation platform integrating a TENG with conductive poly(3,4-ethylenedioxythiophene) (PEDOT)-reduced graphene oxide (rGO) hybrid microfibers has demonstrated significant potential for neural tissue engineering. The PEDOT-rGO scaffold provides excellent electrical conductivity and cytocompatibility, promoting mesenchymal stem cell (MSC) adhesion, proliferation, and neural lineage commitment. The conductive hydrogel scaffold coupled with pulsed electrical stimulation generated by a TENG and powered by biomechanical energy from human walking markedly enhanced MSC neural differentiation without the need for additional biochemical induction factors [34,77]. This strategy highlights the potential of wearable, self-powered bioelectronic systems to provide motion-dependent electrical stimulation, offering a promising approach for stem cell-based nerve regeneration and the development of next-generation implantable neural scaffolds. Thus, TENGs harness passive human biomechanical energy to generate self-powered systems for ES-based therapeutic interventions [34].

Further progress toward controllable transient electroceutical systems was demonstrated by Lee et al., in 2023, who reported an on-demand bioresorbable neurostimulator integrating ultrasound-mediated power delivery with an active elimination mechanism [25]. The system incorporates an acoustically triggerable transient triboelectric nanogenerator (ACT-TENG) composed of PHBV/PEG:ChCl composite membranes and magnesium electrodes (Fig. 4c). When activated by transcutaneous low-intensity ultrasound, the device generates voltage peaks of up to 7.8 V at 40 MΩ, with a reported maximum power density of 102.4 μW cm^−2^, enabling effective neural stimulation. A key innovation of this design lies in its transition from passive degradation, characterized by extremely slow dissolution, to an actively triggered elimination mode induced by high-intensity ultrasound. Under these conditions, the device rapidly loses its energy-generating capability and undergoes mechanical disintegration in vivo within 2 h; fragmentation is distinct from complete biological clearance. In mouse models of nerve compression injury and hereditary neuropathy, daily ultrasound-driven stimulation significantly improved nerve conduction velocities and restored myelination to near-normal levels, highlighting the potential of remotely controllable transient electroceuticals.

Continuing this trend toward performance optimization, Cai et al. recently developed an integrated implantable TENG system for peripheral nerve repair by incorporating hollow mesoporous silica (H-SiO_2_) into a high-dielectric polydimethylsiloxane matrix [26](Fig. 4d). The inclusion of mesoporous silica significantly enhanced the dielectric constant and surface charge density, thereby improving electrical output and enabling the device to generate stable biphasic pulses in response to subtle biological mechanical movements. In a rat sciatic nerve injury model, the TENG-driven electrical stimulation markedly upregulated growth-associated protein 43 (GAP-43) expression and promoted axonal elongation. The stimulation group exhibited significantly higher nerve conduction velocity and improved muscle wet weight ratios compared with untreated controls, indicating effective mitigation of denervation-induced muscle atrophy. This study highlights the potential of mesoporous material-enhanced flexible electronics to further improve the performance of self-powered electroceutical systems. Another study by Niu et al. developed a fully biodegradable silk fibroin (SF) based TENG nanogenerator to improve peripheral nerve regeneration. The device was doped with Li^+^ ions to enhance its electrical performance and to modify its ionic/interfacial polarization, dielectric properties, and electron affinity. The device showed excellent electrical performance, generating an output voltage of 58 V and a current of 6.1 μA. In vivo sciatic nerve model showed that SF-TENG-driven electrical stimulation enhances nerve regeneration and muscle preservation as compared to non-stimulated groups. Histological analysis also showcased 2210.9 ± 653.0 μm^2^ muscle regeneration in the ES-treated group. SF-TENG stimulation promoted remyelination and axonal growth, increasing myelin sheath thickness from 372.7 ± 115.9 nm in controls to 604.2 ± 143.6 nm in the ES group [46]. The key material compositions, electrical outputs, in vivo models, and functional outcomes across these TENG-based platforms are systematically compared in Table 3, which illustrates how each successive design addressed the limitations of its predecessor, from fixed-lifetime biodegradable generators to motion-adaptive hybrid systems and remotely controllable ultrasound-triggered devices. Taken together, these studies illustrate the rapid technological evolution of TENG-based neural interfaces from early biodegradable power sources to motion-responsive, hybrid, and remotely controllable electroceutical platforms, highlighting their growing potential to provide adaptive, battery-free therapeutic stimulation for next-generation neural regeneration therapies [19].

### 4.3 Piezoelectric nanogenerators

PENG–based systems for nerve repair typically employ piezoelectric materials, such as PVDF or PLLA, integrated into fibrous nerve guidance conduits. Material design and operating mechanism are detailed in Section 2.3; the applications below distinguish between local motion and externally supplied mechanical activation. The resulting low-intensity electrical stimulation (ES) mimics the body's natural electrophysiological environment and promotes nerve regeneration by enhancing cellular adhesion, migration, proliferation, and differentiation [31,37]. At the cellular level, these electrical cues activate voltage-gated calcium channels, leading to increased intracellular calcium influx that regulates microtubule organization and the expression of regeneration-related proteins, ultimately facilitating accelerated axonal elongation and functional nerve recovery [11,[20], [21], [22], [23],[28], [29], [30], [31]] [37]. Unlike conventional electrical stimulation that requires exogenous electrodes and wires, biodegradable PENG-based nanogenerators provide temporary, localized electrical cues and may degrade after treatment, provided that the fate of all implanted components is established.

#### 4.3.1 Motion-responsive piezoelectric scaffolds

Early studies established the feasibility of intrinsic motion-driven piezoelectric scaffolds for nerve regeneration. Cheng et al., in 2020 fabricated a three-dimensional porous PVDF/PCL composite nerve guidance conduit via a cast/annealing-solvent displacement method, in which annealing at 55 °C facilitated transformation of PVDF from the α to the piezoelectrically active β phase [30]. The optimized 20% PVDF/PCL formulation exhibited a porosity of 76.2 ± 5.4% (Fig. 5a), a Young's modulus of 67.76 MPa, and a piezoelectric coefficient (d_33_) of 13.2 p.m. V^−1^, a 12-fold enhancement over the PCL control (1.1 p.m. V^−1^). In vitro, rat Schwann cells cultured on the composite scaffolds showed significantly greater neurite extension (median length: 41.9 μm vs. 28.5 μm on PCL) and a 1.9-fold higher proportion of neurite-bearing cells, attributed to transient piezoelectric potentials generated by cell-induced substrate deformation. In a 15-mm rat sciatic nerve defect model at four months post-implantation, the PVDF/PCL group achieved an SFI of −27.7 and an NCV of 35.5 m/s, significantly outperforming PCL controls (−41.5; 14.6 m/s) and closely approaching autograft values (−21.1; 33.7 m/s). Myelin sheath thickness (0.86 μm vs. 0.53 μm) and micro-vessel density (129 mm^−2^ vs. 54 mm^−2^) were both markedly superior to PCL controls (Fig. 5a), consistent with contributions to axonal myelination and VEGF-mediated angiogenesis. Gastrocnemius muscle assessment further confirmed effective attenuation of denervation atrophy, with higher muscle mass (2.94 g vs. 2.22 g) and fewer severely atrophic myofibers (7.6% vs. >20%) compared to controls.

**Fig. 5:**
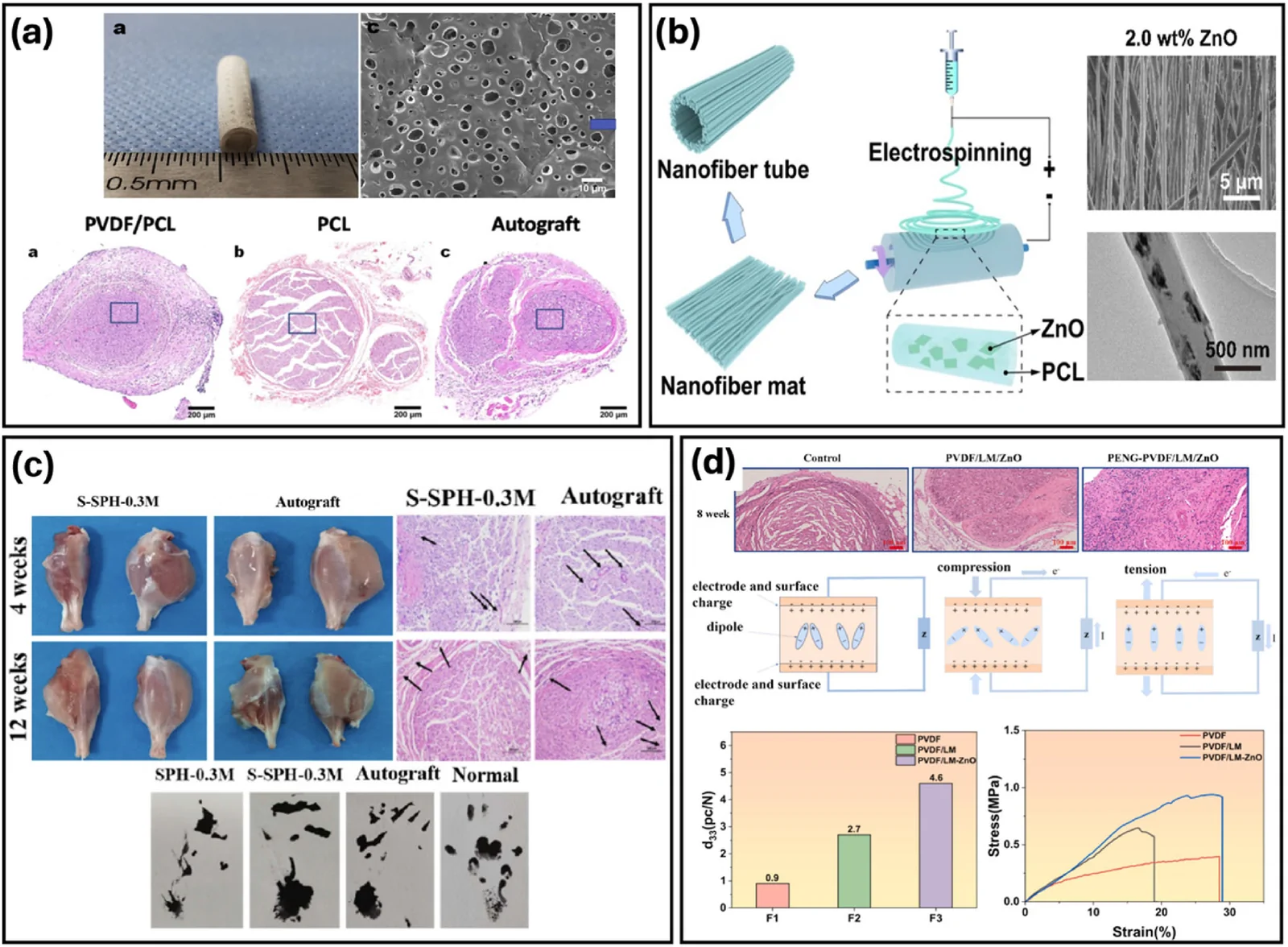
(a) Optical and SEM images (×1000) of the 20% PVDF/PCL scaffold showing its multilayered microporous structure, and H&E staining of regenerated sciatic nerve sections (Reproduced with permission. Copyright © 2020, Elsevier) [30].(b) Electrospun PCL/ZnO nanofiber mat rolled into a nerve conduit (diameter: 0.5 mm) for nerve bridging, with SEM and TEM images of PZNF nanofibers at 2 wt% ZnO (Reproduced with permission. Copyright © 2022, Elsevier) [37].(c) Gastrocnemius muscle morphology, histological evaluation of regenerated nerve, and gait analysis comparing the autograft and exercise-activated groups, comparing exercise-activated stimulation with autograft repair (Reproduced with permission [47]. Copyright © 2023, ACS). (d) H&E staining of peri-conduit tissue at 8 weeks, assessing peri-conduit tissue response in the self-powered group. Schematic of the piezoelectric effect, d_33_ coefficients, and mechanical performance curves of different PVDF-based NGCs (Reproduced with permission. Copyright © 2026, Elsevier) [48].

Building on the principle of endogenous piezoelectric stimulation [37], Mao et al., in 2022 introduced ZnO as an inorganic piezoelectric filler within an electrospun PCL matrix (PZNF), exploiting ZnO's hexagonal wurtzite crystal structure to generate stable electrical output without requiring polymer phase engineering (Fig. 5b). The PZNF conduit exhibited a tensile strength of 10.2 MPa and produced output voltages of 40-120 mV under a reported characterization load (∼0.3 MΩ), with output amplitude scaling positively with ZnO concentration. Biocompatibility was maintained at 2 wt% ZnO, where zinc ion release (∼0.06 mg/L) remained well below the cytotoxic threshold (∼0.63 mg/L). In vitro, Schwann cells subjected to PZNF-coupled mechanical loading via a Flexcell tension system exhibited significantly elevated NGF and VEGF expression at both mRNA and protein levels. In a 10-mm rat sciatic nerve defect model, PZNF-implanted animals achieved functional and morphological recovery at the 4-week assessment; this endpoint does not establish faster recovery than studies with different follow-up schedules, with SFI scores comparable to direct nerve bridging, increased myelin sheath density, and continuous NF200/S100-positive axonal regeneration. Transcriptomic analysis at day 28 identified GRB2 as a putative electrically sensitive hub protein within the RAS/MAPK signaling cascade, whose expression was sharply upregulated after 48 h of piezoelectric stimulation, providing a molecular framework linking endogenous biophysical cues to downstream neurotrophic signaling [37].

Stratton et al., in 2023 [31] developed a bifunctional electrospun scaffold of aligned PCL fibers embedded with ZnO nanoparticles (0–2 wt%) to simultaneously harness Zn ion bioactivity and ZnO piezoelectricity for SC-mediated axon growth. All scaffolds maintained fiber alignment above 90% with less than 6% cumulative Zn release over four weeks, sustaining a low-level ionic environment without cytotoxicity. In SC–DRG co-cultures, the 1 wt% ZnO group yielded axon lengths approximately 30% greater than PCL-only controls, attributed to the combined effects of Zn-mediated neurotrophin upregulation, piezoelectric electrical cueing, and aligned fiber contact guidance; the 2 wt% ZnO concentration, by contrast, was cytotoxic and abolished DRG attachment. Although BDNF levels were elevated across all co-cultures, peaking at 0.5 wt% ZnO, while MBP immunostaining indicative of myelination was detected only on ZnO-free scaffolds, suggesting that ZnO retains SCs in a pro-growth rather than pro-myelinating state and that further optimization is needed to reconcile axon elongation with myelination competence. In the same year, Zhang et al. fabricated an electrospun SF/PVDF-HFP/Ti_3_C_2_T_x_ MXene composite nerve conduit in which MXene incorporation progressively boosted piezoelectric output up to 100 mV, reduced hydrophobicity (contact angle from 120° to 87°), and conferred antibacterial inhibition exceeding 90%. In vitro, ultrasound-driven piezoelectric stimulation upregulated SC expression of GFAP, S100, and NGF by 1.79-, 2.29-, and 1.53-fold, respectively. In a rat 10-mm sciatic nerve transection model, exercise-activated piezoelectric stimulation, using animal locomotion as the mechanical source, yielded SFI values (−33.3 ± 3.8 at 12 weeks), SC density (292 ± 63 per 10^4^ μm^2^), myelin sheath thickness (423.2 ± 26.8 nm), and myelinated axon diameter (2.3 ± 0.5 μm) all comparable to the autograft group (Fig. 5c), supporting exercise-associated stimulation in this model; matched activity controls are needed to separate electrical and rehabilitation effects.

Chen et al. (2026; published online in 2025) developed biodegradable, amino acid-based piezoelectric nerve guidance conduits composed of aligned PCL-(β-Gly) composite nanofibers, fabricated via a one-step electrospinning process. The aligned nanofiber architecture not only provides topographical cues for directional neurite outgrowth but also generates electrical stimulation in response to mechanical deformation; the reported animal treatment used externally supplied vibration from a low-frequency (15 Hz) massage gun. This is an externally activated, battery-free approach; the reported low temperature rise does not establish the absence of mechanical or thermal risk under other settings. Treatment used 30 min of vibration every other day for 2 weeks, with a 12-week follow-up. In a rat model of 10 mm sciatic nerve defects, piezoelectric stimulation enhanced Schwann cell myelination and neurite outgrowth in vitro, and promoted structural nerve regeneration in vivo. Vibration-triggered ES from the PCL-β-Gly conduits restored 96% of compound muscle action potential (CMAP) amplitude and 99% of gastrocnemius muscle wet weight, closely matching autograft-level functional recovery. These findings demonstrate that amino acid–based piezoelectric NGCs offer a promising, self-powered, and biodegradable strategy for peripheral nerve injury repair [43]. A further composite system was reported by Li et al., in 2026 [48], who fabricated a PVDF/LM-ZnO nerve guidance conduit by electrospinning PVDF with gallium-indium liquid metal followed by hydrothermal ZnO nanoflower growth, synergistically elevating β-phase crystallinity to 71.5% and boosting the piezoelectric coefficient d_33_ from 0.9 to 4.6 pC/N (approximately 5.1-fold; Fig. 5d). In vitro, the conduit promoted aligned proliferation and differentiation of Schwann cells, PC12 neurons, and HUVECs along the nanofiber axis, with Western blot confirming significantly upregulated NT-3 expression. An integrated piezoelectric-triboelectric nanogenerator (PTNG) converted physiological motion into ∼2 V electrical output, with PTNG-derived stimulation outperforming conventional external electrical stimulation in axonal elongation. In a rat sciatic nerve transection model, the self-powered group achieved an SFI of −21.2 ± 3.1 at 8 weeks, significantly superior to the non-stimulated conduit group (−30.6 ± 2.1) and suture-only controls (−55.0 ± 3.7), accompanied by elevated MBP, S100, NF200, and Tuj1 expression, collectively demonstrating that biomechanical energy harvesting can sustain autonomous electrical stimulation for peripheral nerve regeneration. The material compositions, electrical outputs, and key in vivo outcomes of these representative intrinsic motion-driven piezoelectric scaffold studies are summarized in Table 4.

#### 4.3.2 Wireless externally-activated piezoelectric systems

Despite these advances, the electrical output of motion-driven PENG scaffolds remains dependent on sufficient mechanical deformation, which may be limited in immobilized tissues or early stages of nerve injury. To address this limitation, recent studies have introduced wireless acoustic activation strategies, in which ultrasound or shockwave stimulation remotely triggers the piezoelectric response of implanted scaffolds. The foundational viability of this approach was demonstrated by Wu et al., in 2022, who developed a fully biodegradable PENG comprising KNN nanowires (50 wt%) embedded in a PHBV/PLLA matrix, reporting approximately 12 V and 36 μA after friction correction under 0.7 W cm^−2^ ultrasound characterization [44]. For treatment of a 15-mm rat sciatic nerve defect, ultrasound at 100 kHz and 0.3 W cm^−2^ was used; ultrasound-driven stimulation yielded CMAP amplitudes (19.3 mV) and SFI values (−41.2) closely matching the autograft standard, while the implanted nanogenerator simultaneously enabled non-invasive EMG-based monitoring of nerve maturation (Fig. 6a). Building on this foundation, Pi et al., in 2023 introduced a sono-electro-mechanical system in which electrospun PCL/PVDF nanotracts (d_33_ = 14.07 p.m./V) were activated by LIPUS to generate localized signals of tens to hundreds of millivolts, upregulating neurotrophic factors (NGF, BDNF, CNTF) in vitro (Fig. 6b) and achieving autograft-comparable functional recovery in an identical 15-mm defect model at 12 weeks. Critically, by decoupling bioelectric delivery from joint movement, this design overcame a fundamental limitation of motion-dependent piezoelectric systems in immobilized injuries. A mechanically distinct but conceptually aligned strategy was reported by Tai et al., who activated electrospun P(VDF-TrFE) nanofibers (500 nm diameter; 200 mVpp output) using an extracorporeal shockwave device rather than implanted ultrasound coupling [27]. Periodic shockwave application (1000 pulses at 4 bars, twice weekly) improved SFI relative to static conduits and achieved nerve reconnection in 4/4 treated animals across a 15-mm defect, compared with 1/4 controls, with organized myelin regeneration across the full gap confirmed by PS-OCT phase retardation analysis.

**Fig. 6:**
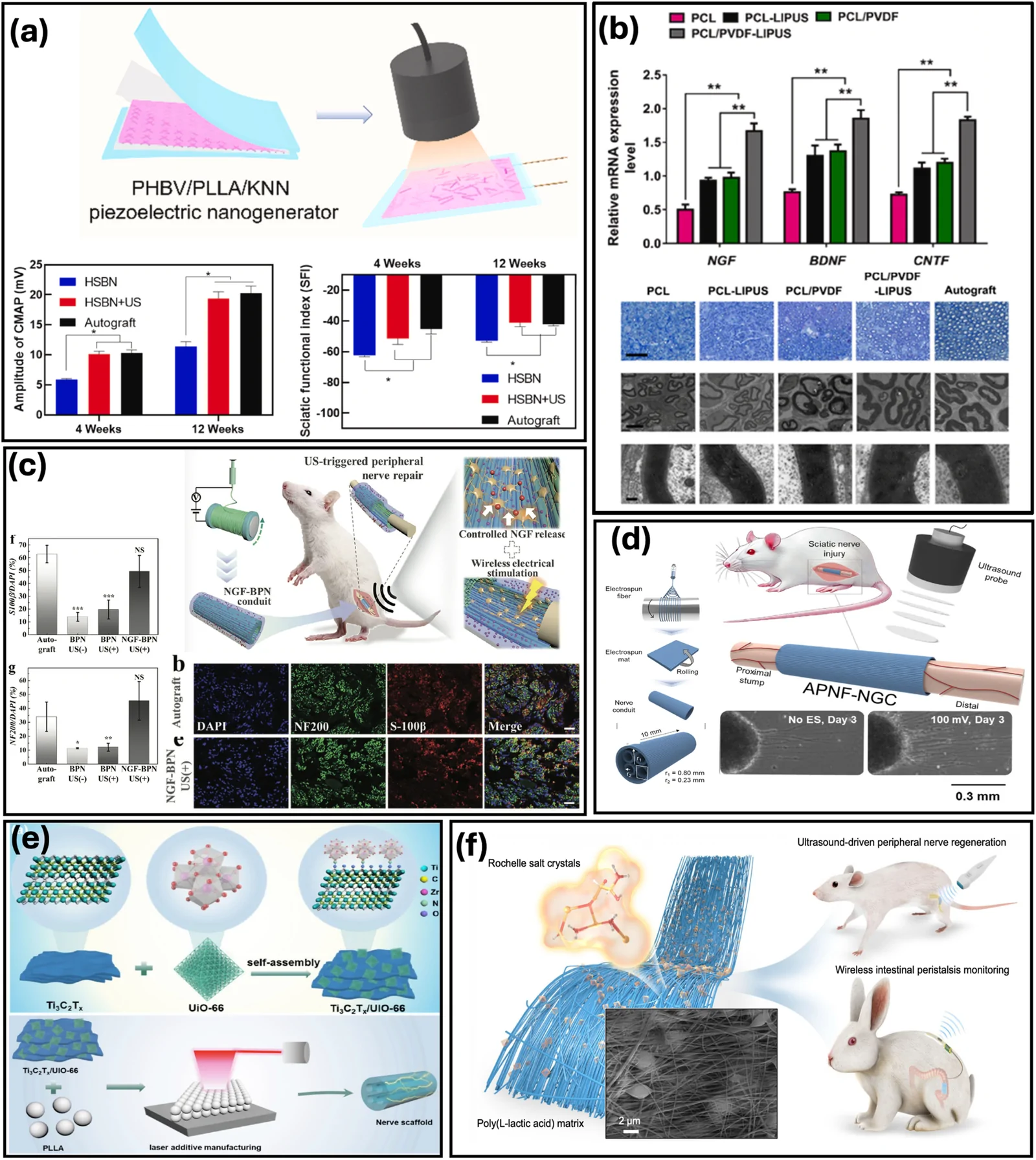
(a) Ultrasound-driven electrical output of the PHBV/PLLA/KNN nanogenerator. Quantitative analysis of CMAP peak amplitudes and SFI values (Reproduced with permission [100]. Copyright © 2022, Elsevier).(b) Effects of sono-electro-mechanical stimulation on Schwann cells and PC12 cells, assessed by relative mRNA expression of NGF, BDNF, and CNTF. Histological analysis of regenerated nerves by toluidine blue staining and TEM (Reproduced with permission [36]. Copyright © 2023, Elsevier).(c) Schematic of NGF-BPN for peripheral nerve regeneration. Immunofluorescence staining of DAPI, NF200, and S-100β in the autograft and NGF-BPN US(+) groups at 8 weeks postoperatively, with quantification of S-100β and NF200 positive areas (Reproduced with permission) [49]. Copyright © 2024, John Wiley and Sons).(d) The ultrasound-driven APNF-NGC, illustrating its four-channel architecture, electrospinning-based fabrication, and proposed mechanism for nerve repair, alongside optical microscopy images of iPSC-derived motor neuron axon elongation with and without ES (Reproduced with permission [50]. Copyright © 2025, John Wiley and Sons).(e) The synthesis process of Ti_3_C_2_T_x_/UIO-66 composite and the fabrication of the nerve scaffold (PLLA) by laser additive manufacturing (Reproduced with permission [28]. Copyright © 2025, ACS).(f) Illustration of PRSC composites enabling ultrasound-driven peripheral nerve regeneration and wireless monitoring of colonic peristalsis, with SEM images of the PRSC morphology (Reproduced with permission [29]. Copyright © 2026, Springer Nature).

Subsequent studies shifted focus from single-modality electrical stimulation toward multi-functional platforms that couple piezoelectric bioelectric cues with complementary topographical, biochemical, or structural guidance. Xu et al., in 2024 reported an ultrasound-responsive NGC (NGF-BPN) featuring an inner layer of BTNPs-doped P(VDF-TrFE) nanofibers (87% alignment; 82.3% β-phase crystallinity; 1.69 V output at 0.75 W/cm^2^) and an outer PNIPAM hydrogel that released NGF via ultrasound-triggered thermal shrinkage [49] (Fig. 6c). This synergistic combination of electrical stimulation and controlled biochemical delivery accelerated recovery across a 10-mm sciatic defect over eight weeks, with SFI values and neural protein expression (S-100β, NF200) showing no significant difference from autograft in the reported comparisons (Fig. 6c), exemplifying a battery-free platform that unifies physical, electrical, and biochemical therapies within a single implant. Complementing this approach, Jeon et al. prioritized biomimetic structural design by arranging aligned PLLA/PEG nanofibers (20 wt% PEG; Young's modulus 128.73 MPa) into a fascicle-mimetic four-channel architecture that generated 161.2 mV (V_pp_) and 58.7 nA (I_pp_) under 40 kHz ultrasound [50]. The resulting APNF-NGC (aligned piezoelectric nanofiber nerve guidance conduit) enhanced axon elongation in iPSC-derived motor neurons in vitro (Fig. 6d) and achieved autograft-comparable reinnervation in an 8-mm rat sciatic defect at 12 weeks, including recovered ankle kinematics (toe-off angle: 64.4 ± 2.6°; contracture angle: 126.6 ± 3.0°) and myelinated axon counts (615 ± 49.9), demonstrating that anisotropic physical guidance and ultrasound-driven stimulation are mutually reinforcing therapeutic axes.

More recent investigations have expanded the mechanistic scope of piezoelectric nerve repair to encompass immune microenvironment modulation and next-generation biodegradable materials with exceptional electromechanical performance. A recent study by Qi et al. developed a suture-free piezoelectric Janus membrane for peripheral nerve regeneration by incorporating aligned GelMA-tannic acid fibers (GT) as the inner layer and methanol-treated silk fibroin (SF) as the outer layer in a band-aid-like fashion. The GT fibers help in tissue adhesion and directional axonal growth, while the SF layer helps in piezoelectric functionality. The membrane exhibited piezoelectric activity under mechanical activation. The fibers showed significantly enhanced PC12 neurite extension and alignment, while also exhibiting antibacterial activity of 94.3% against *Staphylococcus aureus* and 88.5% against *Escherichia coli*. In rat sciatic nerve defect model, the group with SF-GT fiber along with externally activated low-intensity pulsed ultrasound (LIPUS) enhanced axonal regeneration, vascularization, and motor and sensory recovery as compared to the group without stimulation. This was further supported by immunohistochemical analysis showcasing GAP43 and NCAM1 expression, especially after LIPUS activation [51]. Ding et al., in 2025 fabricated a laser-additively manufactured PLLA scaffold incorporating Ti_3_C_2_Tx MXene nanosheets and UIO-66 MOF nanoparticles (Fig. 6e), wherein ultrasonic stimulation exploited the synergy between UIO-66-generated piezoelectric potential and MXene's charge-transfer efficiency to produce a stable peak current of 0.036 μA. This output drove macrophage M1-to-M2 polarization (CD206^+^ cells: 91.02% vs. 78.15% in PLLA controls) by upregulating ATP-sensitive K^+^ channels and suppressing the Ca^2+^-CaMKII-NF-κB axis via VGCC downregulation, while simultaneously promoting BMSC neuronal differentiation (Tuj1 and MAP2 upregulation) in an inflammatory environment, supporting an in vitro immune-related mechanism; this study did not establish functional repair in an animal nerve-gap model. Extending the material frontier, Dai et al., in 2026 embedded Rochelle salt micro-crystals within electrospun PLLA nanofibers and applied uniaxial compression to yield a biodegradable composite (PRSC, Fig. 6f) with a d_33eff_ of 43.1 ± 3.0 pC N^−1^ and g_33_ of 1909.2 mV m N^−1^ [29], surpassing all previously reported biodegradable flexible piezoelectrics [85,117,118]. In a 10-mm rat sciatic defect model, ultrasound-activated PRSC scaffolds achieved an axon length of 7.53 ± 0.18 mm and SFI recovery without a significant difference from autograft in the reported comparisons by modulating intracellular calcium via Cav3.2 channels and upregulating Gap43 and Galanin. Beyond nerve repair, the same material platform functioned as a biodegradable strain sensor (sensitivity: 0.049 ± 0.003 V kPa^−1^) for real-time wireless monitoring of intestinal motility, illustrating the broader translational potential of high-performance biodegradable piezoelectrics across neuromodulation and physiological sensing applications. The key material parameters, stimulation strategies, and in vivo outcomes of these wireless externally activated piezoelectric systems are collectively summarized in Table 5.

Collectively, these studies highlight the rapid technological evolution of piezoelectric electroceutical platforms for nerve regeneration, progressing from early motion-responsive scaffolds that generate endogenous electrical cues, to advanced composite materials with enhanced electrical output, and ultimately to remotely activated acousto-piezoelectric systems capable of delivering externally adjustable electrical stimulation whose delivered dose still requires measurement and multifunctional therapeutic effects. This progression underscores the growing potential of self-powered bioelectronic scaffolds to bridge critical nerve gaps, modulate the regenerative microenvironment, and provide next-generation strategies for peripheral nerve repair.

## 5 Future perspectives and conclusions

Three decades of incremental progress in passive nerve conduits have yielded a frustrating ceiling: hollow tubes and collagen scaffolds reliably bridge short gaps but consistently fail to restore the electrophysiological fidelity that meaningful motor and sensory recovery demands. The past decade's pivot toward self-powered bioelectronic neuromodulation has fundamentally reframed this problem, shifting the design question from “how do we guide axons?” to “how do we recreate the endogenous bioelectric landscape that drives regeneration?” Galvanic cells, triboelectric nanogenerators, and piezoelectric nanogenerators each represent a distinct answer to that question, and their comparative strengths and limitations now define the frontier of the field.

Of the three strategies, self-electrified galvanic devices offer a relatively simple strategy for movement-independent postoperative stimulation. They require no patient compliance, no external hardware, and no activity-dependent mechanical deformation. They simply activate upon contact with physiological fluids and deliver continuous DC stimulation while reactants and electrical continuity remain available. This simplicity is also their most significant constraint: output profile, stimulation duration, and degradation kinetics are largely fixed by material choice at the time of fabrication, leaving little room for post-implantation adjustment [119,120]. The transition from magnesium-to zinc-based anodes has meaningfully extended operational windows and reduced hydrogen evolution, and architectures that couple galvanic stimulation with integrated neural recording provide a basis for future responsive therapy, but recording alone does not establish a closed loop. Nevertheless, the field has yet to demonstrate reliable stimulation delivery calibrated to the weeks-long regeneration timeline in large-animal or human nerve injury contexts, and this remains the central translational bottleneck.

TENGs occupy an intriguing middle ground. They can generate substantially higher voltages than galvanic cells and are inherently synchronized with the patient's own physiological movements, creating physiologically coupled neuromodulation rather than validated therapeutic feedback. Their susceptibility to output variability under immobilization, however, is a practical limitation that restricts their applicability precisely in the acute postoperative phase when stimulation is most critical [4,11,24,26,121]. Hybrid [85] TENG-PENG architectures and acoustically triggered transient devices have begun to address this vulnerability, but the engineering complexity of ensuring stable, therapeutically adequate output across diverse patient activity levels remains unresolved. The most compelling near-term trajectory for TENGs may lie not in isolated nerve repair applications, but in their integration into wearable or partially implantable systems that simultaneously harvest energy from broader body kinematics, including respiratory motion and gait cycles, while delivering targeted stimulation to peripheral injury sites.

PENGs, particularly those that combine ultrasound activation with aligned nanofiber scaffolds, offer versatile options for activation and scaffold design. Driving piezoelectric output externally via ultrasound can reduce the immobilization constraint that limits both TENGs and motion-dependent PENGs, while allowing adjustment of the external activation settings. The recent emergence of fully biodegradable piezoelectric composites with performance metrics approaching those of non-degradable materials marks a materials science inflection point: for the first time, high-output, bioresorbable piezoelectric scaffolds are within reach, offering alternatives to PVDF-containing scaffolds while leaving long-term degradation-product safety to be established. This mechanistic insight that ultrasound-driven piezoelectric stimulation modulates intracellular calcium through defined channel subtypes rather than through a diffuse or poorly characterized pathway, which also provides a rational basis for stimulus parameter optimization that has been largely absent from earlier empirical approaches.

Looking forward, the convergence of these three technologies within integrated, multifunctional platforms may ultimately prove more powerful than any single strategy pursued in isolation. A device architecture that combines the sustained baseline stimulation of a galvanic cell with the on-demand, adjustable output of an acoustically activated piezoelectric layer—while incorporating real-time electrophysiological recording—addresses the full temporal arc of peripheral nerve regeneration. Advances in biodegradable soft electronics, machine learning-assisted signal processing, and biofabrication may enable such closed-loop bio-electroceuticals, but clinical validation remains necessary (Fig. 7). Realizing this translational roadmap will require continued materials innovation, standardized preclinical evaluation frameworks, and regulatory pathways tailored for devices whose functional lifetime is intentionally finite. Ultimately, the foundational science reviewed here establishes that self-powered electrical stimulation can meaningfully accelerate nerve repair; the work ahead lies in translating that proof of concept into a reliable, clinically deployable therapy.

**Fig. 7:**
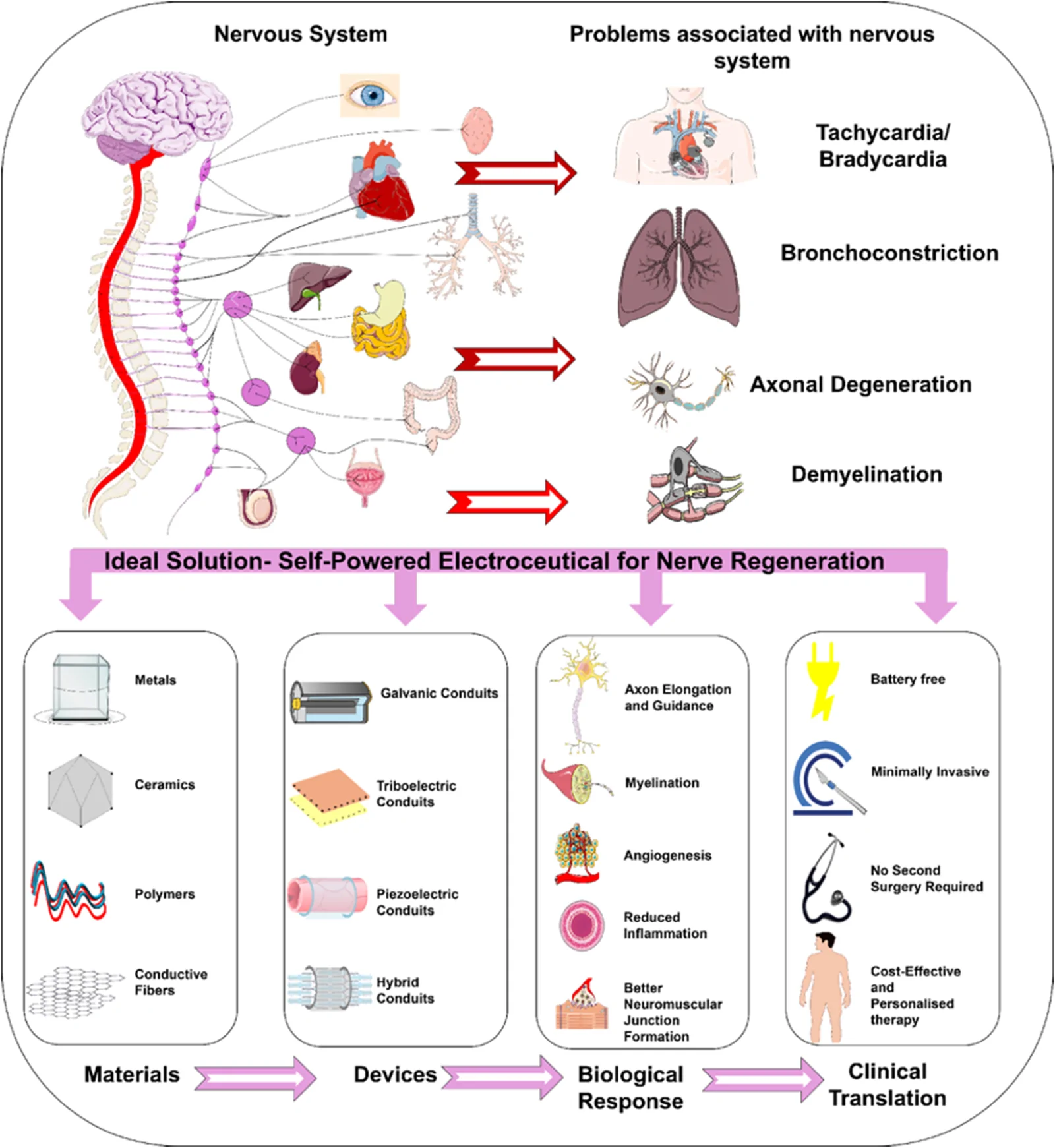
Roadmap from engineered biomaterials to self-powered bioelectroceuticals for nerve regeneration. The original conceptual scheme links materials, energy conversion, and therapeutic applications.

### 5.1 Clinical readiness, regulation, and scalability

Despite encouraging preclinical results, clinical translation of self-powered bioelectronic devices for peripheral nerve regeneration remains limited: most galvanic devices, TENGs, and PENGs have been validated only in small-animal models, with little evidence for long-term performance under clinically relevant anatomical and biomechanical conditions. Translation will require reproducible electrical output, stable therapeutic performance throughout the intended treatment period, predictable material degradation, and validation in large-animal models (pig, sheep, non-human primate) to establish implant stability, anatomical fit, and chronic immune response ahead of first-in-human study [41,122,123].

Biological evaluation of biodegradable bioelectronic devices should follow ISO 10993 [124] at the whole-device level, thereby addressing degradation products, residual materials, sterilization effects, electrical safety, and long-term functionality, rather than individual material properties in isolation. Externally activated systems additionally require systematic validation of acoustic exposure, thermal effects, cavitation, and stimulation reproducibility under clinically relevant conditions [115,[125], [126], [127]]. Beyond biological safety, translation depends on establishing standardized stimulation-dose benchmarks that correlate electrical parameters (current density, charge density, pulse width, frequency, treatment duration) with molecular, histological, electrophysiological, and functional outcomes a framework that would allow direct comparison across galvanic, triboelectric, and piezoelectric platforms and thereby accelerate regulatory approval and commercialization [41,[128], [129], [130]].

### 5.2 Chronic inflammation, material fate, and large-animal validation

All implantable biodegradable bioelectronic devices still elicit a foreign body response that can compromise host and immune responses [131]. Protein adsorption, acute and chronic inflammation, macrophage activation, foreign body giant cell (FBGC) production, fibroblast recruitment, and fibrotic capsule creation are the successive stages of the foreign body response. Excessive fibrosis and collagen deposition increase the distance between the electrode and regenerating nerve tissues, elevating electrical impedance and lowering stimulation efficiency. Therefore, long term evaluation should include serial assessment of macrophage phenotype, FBGC formation, fibrotic capsule thickness, electrophysiology, local tissue microenvironment, degradation, and systemic clearance. Short-term cytocompatibility or a single organ-histology time point cannot establish long-term safety. Studies should span implantation, active treatment, loss of function, maximum degradation burden, and subsequent clearance or stable retention.

Galvanic nerve stimulators, especially Mg, Zn, FeMn, Mo, and Si containing systems, are primarily limited by electrochemical degradation. Local corrosion, pH elevation, gas generation, and systemic distribution may influence macrophage recruitment and tissue compatibility [132]. Consequently, degradation kinetics, electrochemical stability, and systemic clearance should be evaluated to minimize chronic inflammation while maintaining stable electrical output. In TENGs, chronic inflammation is largely associated with repeated mechanical deformation, micromotion at the implant-tissue interface, and incomplete degradation of structural polymers or conductive materials. These contribute to sustained macrophage activation and promote fibrotic remodelling and escalate collagen capsule thickness, thereby reducing electrical coupling between the device and regenerating nerve tissue [133]. Moreover, in PENGs, chronic inflammatory responses are governed by the incomplete degradation profile of piezo polymers and fillers. Biodegradable polymers such as silk fibroin, PLLA, and PLGA exhibit good mechanical compatibility, incomplete degradation or prolonged retention of PVDF, PDMS, persistent conductors, and ceramic or carbon-based fillers in the surrounding matrix may sustain macrophage activation, inflammasome signaling, and fibrotic encapsulation. Therefore, biological evaluation should include complete assessment of device degradation, systemic clearance, and long-term tissue compatibility using a risk-based framework [132].

Large-animal validation remains a major translational gap for bioelectronic devices: galvanic, TENGs, and PENGs technologies. Transmission through excised porcine/rabbit/ tissue is not equivalent to regeneration in a living large-animal nerve. Most studies have been performed in rodent models, which do not adequately reproduce clinically relevant nerve diameter, regeneration distance, delayed repair and movement, chronic inflammation, or immune responses encountered in humans. Consequently, the long-term electrical performance studies should be performed to understand the degradation behavior, chronic inflammation, fibrotic encapsulation, and material fate of these devices following established standard protocol (ISO10993) [124]. Future models should reflect clinically relevant nerve dimensions, delayed repair, and physiological movement, and include autograft and matched non-stimulating controls. Outcome measures should combine motor and sensory recovery, muscle force, electrophysiology, nerve organization, chronic inflammatory responses, degradation, and systemic clearance of implanted materials. A proposed 6-12 month observation period may be appropriate for some models, with extension for slow degradation or retained components; this is a development target, rather than a universal regulatory requirement.

### 5.3 Closed-loop control and real-time feedback

Closed-loop bioelectronic systems represent the next generation of neural interfaces by integrating energy harvesting, sensing, signal processing, and adaptive stimulation into a single autonomous platform. A therapeutic closed loop requires a measured variable, a target or decision rule, an actuator that changes stimulation, and evidence that this adjustment improves dose control or outcome; physiological coupling and simultaneous neural recording, as in FI-NES and Bio-Restor, do not establish that complete loop. Candidate feedback variables include delivered current/charge, electrode impedance, evoked compound muscle action potential (CMAP), mechanical deformation at the electrode–tissue interface, and other validated neural responses [52,134]. Impedance monitoring is highly sensitive to interface changes but is not a specific measure of nerve regeneration. Practical implementation additionally requires stimulation-artifact rejection, defined sensing intervals, bounded energy budgets, and safe shutdown during signal loss or excessive polarization. The engineering solution is platform-specific: ultrasound-driven systems can adjust burst timing or source intensity, whereas galvanic systems require added switching or current-limiting hardware. Validation should directly compare open-loop and feedback-controlled treatment under matched resources, including disturbances and progressive degradation, to establish whether adaptive control meaningfully improves outcomes.

Future biodegradable bioelectronic platforms aim to combine self-powered energy harvesting, multimodal sensing, wireless communication, and adaptive stimulation within a single implantable device. Realizing this will require low-power sensing electronics, robust artifact rejection, and energy-efficient control algorithms operating within the limited power budget of biodegradable self-powered systems. Candidate technologies include galvanic self-powered stimulators, triboelectric and piezoelectric nanogenerators, ultrasound-mediated neuromodulation, and magnetoelectric interfaces. Translation will require systematic improvement in material biodegradability, electrical performance, and closed-loop control algorithms, progressing from in vitro and small-animal studies to validation in clinically relevant large-animal models, with attention to implantation stability, immune response, degradation behavior, and long-term wireless communication reliability. Achieving this alongside scalable manufacturing and regulatory compliance would allow biodegradable electroceuticals to mature into minimally invasive, energy-autonomous platforms for precision peripheral nerve regeneration.

### 5.4 Proposed validation milestones

The schematic illustrates the bioengineering-to-clinical-practice pipeline for self-powered bioelectronics in peripheral nerve regeneration. Peripheral nervous system injury impairs neural connections, which leads to clinical disorders such as cardiac rhythm abnormalities, bronchoconstriction, axonal degeneration, and demyelination. To overcome conventional therapy limitations, self-powered electroceutical platforms integrate biodegradable electroactive materials that will effectively produce implantable bioelectronic devices capable of generating therapeutic electrical stimulation.

The roadmap begins with the selective electroactive materials selection, including biodegradable metals, polymers, ceramics, and conductive fibers, which determine the electrical, mechanical, and degradation characteristics of the device. The implantable materials are engineered as galvanic, triboelectric, piezoelectric, and hybrid nerve guidance conduits, where material composition, device architecture, and energy-transduction mechanisms collectively regulate electrical output and stimulation duration by converting endogenous electrochemical or biomechanical energy into therapeutic electrical output. Clinically optimized electrical cues will activate multiple regenerative processes, including Schwann cell activation, axonal elongation, remyelination, angiogenesis, neuromuscular junction formation, and modulation of inflammatory responses, ultimately promoting functional peripheral nerve regeneration. At the translational level, biodegradable self-powered platforms offer several potential clinical advantages, including battery-free operation, minimally invasive implantation, elimination of secondary removal surgery, and personalized neuromodulation.

## CRediT authorship contribution statement

**Fu-Cheng Kao:** Writing – review & editing, Writing – original draft, Investigation, Data curation, Conceptualization. **Monika Pebam:** Writing – review & editing, Writing – original draft, Visualization, Investigation, Data curation, Conceptualization. **Parag Parashar:** Writing – original draft, Investigation, Formal analysis, Data curation. **Sheng-Fen Wang:** Writing – review & editing, Investigation, Data curation. **Ping-Yeh Chiu:** Writing – review & editing, Formal analysis. **Tsung-Ting Tsai:** Writing – review & editing, Visualization. **Chi-Chien Niu:** Writing – review & editing, Investigation, Data curation. **Shih-Heng Chen:** Writing – review & editing, Investigation, Formal analysis. **Dongwhi Choi:** Writing – review & editing, Supervision, Conceptualization. **Ming-Kai Hsieh:** Writing – review & editing, Supervision, Project administration, Conceptualization. **Zong-Hong Lin:** Writing – review & editing, Supervision, Resources, Project administration, Funding acquisition, Conceptualization.

## Declaration of competing interest

The authors declare the following financial interests/personal relationships which may be considered as potential competing interests: Zong-Hong Lin reports financial support was provided by National Science and Technology Council. If there are other authors, they declare that they have no known competing financial interests or personal relationships that could have appeared to influence the work reported in this paper.

## Data Availability

No data was used for the research described in the article.
